# The role of aging and brain‐derived neurotrophic factor signaling in expression of base excision repair genes in the human brain

**DOI:** 10.1111/acel.13905

**Published:** 2023-06-19

**Authors:** Sofie Lautrup, Camilla Myrup Holst, Anne Yde, Stine Asmussen, Vibeke Thinggaard, Knud Larsen, Lisbeth Schmidt Laursen, Mette Richner, Christian B. Vægter, G. Aleph Prieto, Nicole Berchtold, Carl W. Cotman, Tinna Stevnsner

**Affiliations:** ^1^ Department of Molecular Biology and Genetics Aarhus University Aarhus Denmark; ^2^ Department of Clinical Molecular Biology University of Oslo and Akershus University Hospital Lørenskog Norway; ^3^ Department of Biomedicine, Danish Research Institute of Translational Neuroscience – DANDRITE, Nordic EMBL Partnership for Molecular Medicine Aarhus University Aarhus Denmark; ^4^ Institute for Memory Impairments and Neurological Disorders University of California Irvine California USA; ^5^ Instituto de Neurobiología UNAM‐Juriquilla Juriquilla Mexico

**Keywords:** aging, base excision repair, brain, brain‐derived neurotrophic factor, cyclic‐AMP response element‐binding protein, DNA repair, neurons

## Abstract

DNA damage is a central contributor to the aging process. In the brain, a major threat to the DNA is the considerable amount of reactive oxygen species produced, which can inflict oxidative DNA damage. This type of damage is removed by the base excision repair (BER) pathway, an essential DNA repair mechanism, which contributes to genome stability in the brain. Despite the crucial role of the BER pathway, insights into how this pathway is affected by aging in the human brain and the underlying regulatory mechanisms are very limited. By microarray analysis of four cortical brain regions from humans aged 20–99 years (*n* = 57), we show that the expression of core BER genes is largely downregulated during aging across brain regions. Moreover, we find that expression of many BER genes correlates positively with the expression of the neurotrophin brain‐derived neurotrophic factor (BDNF) in the human brain. In line with this, we identify binding sites for the BDNF‐activated transcription factor, cyclic‐AMP response element‐binding protein (CREB), in the promoter of most BER genes and confirm the ability of BDNF to regulate several BER genes by BDNF treatment of mouse primary hippocampal neurons. Together, these findings uncover the transcriptional landscape of BER genes during aging of the brain and suggest BDNF as an important regulator of BER in the human brain.

## Graphical abstract

DNA damage is a central contributor to the aging process. We uncover the transcriptional landscape of base excision repair (BER) genes during aging of the human brain and find a general downregulation with age. Our results suggest BDNF, which itself is reduced with aging, as an important regulator of BER. One way, BDNF may stimulate expression of genes encoding BER proteins could involve cyclic‐AMP response element binding protein‐induced transcription.

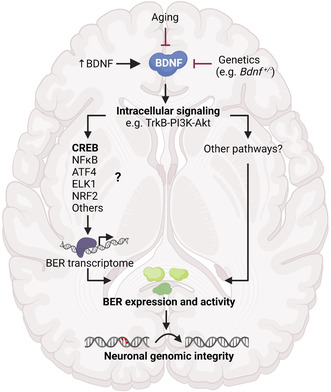

**Abbreviations**
AP: apurinic/apyrimidinic
APE1: AP endonuclease 1
BDNF: brain‐derived neurotrophic factor
BER: base excision repair
CRE: cAMP‐responsive elements
CREB: cyclic‐AMP response element binding protein
DSBR: double‐strand break repair
EC: entorhinal cortex
EMSA: electrophoretic mobility shift assay
FEN1: flap endonuclease 1
HC: hippocampus
LIG1: DNA ligase I
LIG3: DNA ligase III
LP: long‐patch
NER: nucleotide excision repair
PCG: postcentral gyrus
PCNA: proliferating cell nuclear antigen
PNKP: polynucleotide kinase/phosphatase
POLB: DNA polymerase β
ROS: reactive oxygen species
SFG: superior frontal gyrus
SP: short patch
TrkB: tropomyosin receptor kinase B
TSS: transcription start site
XRCC1: X‐ray cross complementing group 1

## 1 INTRODUCTION

Aging and age‐related neurodegenerative diseases are associated with the accumulation of oxidative DNA damage as well as reduced DNA repair activity in the brain (Akbari et al., 2015; Leandro et al., 2015). Due to the continuous electrochemical signaling between neurons, substantial amounts of cellular energy are required. During the cellular production of ATP reactive oxygen species (ROS) are released by the mitochondrial electron transport chain. Hence, high ATP production results in high ROS production, which can lead to oxidative damage of various cellular macromolecules including DNA, proteins, and lipids in the mitochondria but also in other cellular compartments such as the nucleus. If left unrepaired, these damages can lead to cellular dysfunction and eventually trigger cell death. Due to their post‐mitotic status and relatively low levels of antioxidant defenses, neurons rely heavily on their DNA repair capacity for maintaining DNA integrity. The base excision repair (BER) pathway is the primary pathway responsible for the repair of oxidative lesions both in the nuclear and mitochondrial DNA. Although not completely equivalent, both nuclear and mitochondrial BER are central in the maintenance of genome stability in neurons (Sykora et al., 2013; Wang et al., 2017).

The BER pathway is initiated when a lesion‐specific DNA glycosylase recognizes and excises a damaged base, leaving an apurinic/apyrimidinic (AP) site in the DNA. Incision of the AP site is then performed by either a bifunctional DNA glycosylase, followed by end processing by AP endonuclease 1 (APE1) or polynucleotide kinase/phosphatase (PNKP), or the AP site is cleaved by APE1, if a monofunctional DNA glycosylase recognized the base lesion. BER has two sub‐pathways, short‐patch BER (SP‐BER) and long‐patch BER (LP‐BER). In SP‐BER, DNA polymerase β (POLB), in complex with X‐ray cross complementing group 1 protein (XRCC1), inserts one nucleotide followed by ligation by DNA ligase I or III (LIG1 or LIG3). In LP‐BER, DNA polymerase δ/ε in complex with proliferating cell nuclear antigen (PCNA) or POLB, displaces a longer stretch of nucleotides, followed by flap removal by flap endonuclease 1 (FEN1) and ligation by LIG1 (Krokan & Bjoras, 2013). In the post‐mitotic neurons, POLB is the main polymerase in both SP‐ and LP‐BER (Sykora et al., 2013).

A number of studies have described changes in the amount and/or activity of BER enzymes during normal brain aging. However, most studies have been conducted in rodents (Cabelof et al., 2002; Chen et al., 2002; Gredilla et al., 2010, 2012; Imam et al., 2006; Swain & Rao, 2012), while changes during normal aging in the human brain are largely unknown. Moreover, common for most studies is that the analyses have been restricted to a few selected BER genes. To our knowledge, a comprehensive analysis of age‐associated changes in the expression of the collective battery of core BER genes in the human brain is still missing.

Besides an incomplete understanding of BER expression patterns in the aging human brain, insights into how the BER pathway is regulated in the brain and the regulatory mechanisms that may contribute to age‐related changes are sparse. Yang et al. has suggested APE1 to be regulated by the neuronal growth factor brain‐derived neurotrophic factor (BDNF) via the transcription factor cyclic‐AMP response element binding protein (CREB) in cortical rat neurons and in mouse cortex and hippocampus (HC) as a response to a short‐term period of aerobic exercise (Yang et al., 2014). Furthermore, neuronal expression of APE1 and POLB is regulated by CREB in response to different types of stimuli (Pei et al., 2019; Stetler et al., 2010; Yang et al., 2010, 2016).

Via transcription factors including CREB, BDNF signaling is a master regulator of synaptic plasticity, neurogenesis, and neuronal protection. BDNF is involved both in the developing and adult central nervous system, where it is produced by neurons as a pro‐peptide (proBDNF), which is proteolytically cleaved to mature BDNF. BDNF binds the high‐affinity cell‐surface receptor tropomyosin receptor kinase B (TrkB) causing its activation and thereby stimulation of several intracellular signaling cascades, such as the phosphatidylinositol pathway. This leads to the activation of specific transcription factors and expression of genes involved in vital processes in the nervous system (Reichardt, 2006). CREB is activated by phosphorylation at a conserved serine leading to recruitment of its transcriptional co‐factor CREB‐binding protein and subsequently the transcription machinery to the promoter of CREB target genes (Mayr & Montminy, 2001). The activity of the BDNF/TrkB/CREB regulatory axis has been reported to decline with age in the brain (Hattiangady et al., 2005; Paramanik & Thakur, 2013; Romanczyk et al., 2002; Webster et al., 2006; Yamamoto‐Sasaki et al., 1999). Thereby, the BDNF axis can potentially affect the expression of a large number of its target genes during aging, as observed for many synaptic genes downstream of BDNF (Berchtold et al., 2013; Oh et al., 2016).

Here, we perform an exhaustive analysis of the expression profile of core BER genes during human brain aging as well as examine whether the BDNF signaling pathway contributes to regulation of BER in the brain. We show that the expression of the majority of BER genes is downregulated during aging in four different human brain regions. Moreover, we demonstrate that the expression of many of the BER genes displays a positive correlation with BDNF levels in the human brain suggesting that BDNF works as an important regulator of BER. Based on these findings, we explore whether there is a causal link between the BDNF axis and BER. We show that the promoter region of a large fraction of the BER genes contains potential CREB‐binding sites and CREB binds to the majority of these sites in vitro. Using primary mouse hippocampal neurons, we determine the effect of BDNF treatment on protein levels and activity of selected BER enzymes and observe a stimulatory effect on both NEIL2, APE1, and POLB. In addition, we examine the effects of reduced BDNF levels, as seen in *Bdnf* heterozygous mice, on the DNA repair capacity in mouse brain.

## 2 MATERIALS AND METHODS

### 2.1 Microarray analysis

Frozen post‐mortem human brain samples were obtained from 22 young individuals (age 20–59 years) and 33 aged individuals (69–99 years) from seven well‐established National Institute on Aging Alzheimer's disease brain banks (Table S1). Details regarding inclusion criteria, RNA extraction and purification, and microarray analysis can be found here (Berchtold et al., 2008, 2013, 2019).

Probe sets used in the microarray analysis are listed in Table S2. For genes with more than one probe set an average expression was calculated. Selected subsets of age‐affected genes determined by microarray analysis have been validated by qPCR demonstrating a very high agreement between the two methods (Berchtold et al., 2008, 2013). Microarray data are available in the Gene Expression Omnibus database (www.ncbi.nlm.nih.gov/geo) with accession number GSE11882.

### 2.2 In silico CREB‐binding predictions

To predict putative CREB‐binding sites in the promoter regions the Salk Institute CREB target database (Zhang et al., 2005) was used. The recommended threshold for the ChIP‐on‐chip database was set at a binding ratio >2 and a *p*‐value <0.001, although a smaller binding ratio cannot rule out the possibility of binding between CREB and the CRE site (sensitivity with selected cutoffs is 50%; Zhang et al., 2005). In addition, the Champion ChIP Transcription Factor Search Portal (DECODE database, Qiagen) was used to compare the findings from the Salk Institute CREB target database to another database.

### 2.3 Animals and cells

*Bdnf* ^+/−^ and *Bdnf* ^+/+^ littermate controls (Ernfors et al., 1994) were kindly provided by Prof. A. Nykjær (Department of Biomedicine, Aarhus University, Denmark). *Bdnf* ^*−/−*^ mice die during the second postnatal week due to gross neuronal developmental defects, and therefore they were not included in this study (Ernfors et al., 1994). The strain used was backcrossed at least 10 generations into C57BL/6JBomTac (Taconic, Denmark) before use. Primary hippocampal neurons were obtained from P0 mice. All experiments were approved by the Danish Animal Experiments Inspectorate under the Ministry of Justice (Permit 2012‐15‐2035‐00007 and 2016‐15‐0202‐00051) and carried out according to institutional and national guidelines. All animals were bred and housed at the Animal Facility at Aarhus University, Denmark. Animals were housed in groups of up to five mice per cage (42 × 25 × 15 cm) under pathogen‐free conditions with a 12‐h light/12‐h dark schedule and fed standard chow and water ad libitum. Mice (4‐months old) were sacrificed by cervical dislocation and brain regions isolated in PBS on sterile Sylgaard platforms. After dissection, tissues were immediately frozen on dry ice‐EtOH slurry.

### 2.4 Nuclear extracts for gel shift assay

Nuclear protein extract from mouse brain was isolated essentially as previously described (Lahiri & Ge, 2000; Unnikrishnan et al., 2009). Briefly, 500 μL ice‐cold buffer A [10 mM Hepes‐KOH pH 7.9, 10 mM KCl, 0.1 mM EDTA, 0.1 mM EGTA, 1 mM DTT, and 1% protease inhibitor cocktail, phosphatase inhibitor cocktail 2, and phosphatase inhibitor cocktail 3 (Sigma)] was added to 100 mg tissue followed by five pestle strokes. After addition of NP‐40 (final conc. 0.5%) five additional strokes of homogenization were performed. After 10 min incubation at 4°C samples were centrifuged for 1 min at 4000*g*. Three hundred microlitre ice‐cold buffer C (20 mM Hepes‐KOH pH 7.9, 400 mM NaCl, 1 mM EDTA, 1 mM EGTA, 1 mM DTT and 1% protease inhibitor cocktail, phosphatase inhibitor cocktail 2, and phosphatase inhibitor cocktail 3) was added to the nuclear pellets and the samples were vortexed and then rotated for 15 min at 4°C. Then, samples were centrifuged for 5 min at 11,000*g* and the supernatant was dialyzed for 4 h at 4°C in Slide‐A‐Lyzer MINI Dialysis Units (cut off 7000 MWCO; Thermo Scientific) against 1 L dialysis buffer (20 mM Tris–HCl pH 8, 100 mM KCl, 5% glycerol, 0.1 mM DTT, 0.1 mM PMSF, and 1 mM NaF). Protein concentration was determined by Bradford protein assay.

### 2.5 5′ ƴ‐ATP labeling of DNA–oligomers

One hundred nanogram purified single‐stranded DNA oligomer was incubated with ^32^P‐ƴ‐ATP and T4 Polynucleotide kinase (Thermo Scientific) in forward buffer A (Thermo Scientific) for 90 min at 37°C, followed by 1 min incubation at 95°C. A G50 column (BioRad) was used for removal of free radioactive phosphates. One hundred millimolar EDTA, 175 mM KCl and 400 ng complementary unlabeled oligomer was added to the eluate. The sample was boiled for 5 min and cooled down overnight (ON). Full annealing between labeled and unlabeled oligomer was confirmed on a 20% native polyacrylamide gel. After electrophoresis, labeled oligomers were visualized using phosphor storage screens (Amersham Bioscience) and a Typhoon FLA 9500 scanner (GE Healthcare). The amount of labeled DNA lost on the column was quantified by use of Image Quant software.

### 2.6 Electrophoretic mobility shift assay (EMSA)

DNA‐binding reactions were performed in a volume of 20 μL. Nuclear extract (10 μg) was incubated for 60 min at 4°C in binding buffer (100 mM Tris–HCl, 500 mM KCl, 10 mM DTT, 2.25% glycerol, 5 mM MgCl_2_, 0.05% NP‐40, 50 ng/μL poly‐dI‐dC, 0.05 mg/mL salmon sperm DNA, 10 μg/mL BSA). In super shift reactions, anti‐CREB antibody (Cell signaling, #9197) was added (0.3 μL). Then, 40 fmol 5′‐radiolabeled double‐stranded oligonucleotides were added, and incubation was continued at room temperature (RT) for 20 min. Oligonucleotides corresponded to in silico predicted CRE sites and immediate up‐ and downstream flanking promoter regions of selected BER genes (Table S3). In competitive reactions, 50‐fold excess of unlabeled oligonucleotide corresponding to the promoter region of POLB containing a previously established CRE site was added to the reaction mixture. Subsequently, glycerol was added to a final concentration of 5%, and samples were resolved on a 5% native polyacrylamide gel in 0.5× TBE buffer (70 V for 5 h at 4°C).

### 2.7 Isolation of primary mouse hippocampal neurons and treatment with recombinant BDNF

Brains were isolated from early postnatal mice (P0). All meninges were carefully removed, and hippocampi were isolated and transferred to cold Leibovitz's L‐15 medium (Life Technologies). After 3 min centrifugation at 2000*g*, the pellet was digested with papain solution (L‐15 medium, 2 mM EDTA, 20 U/mL Papain (BioNordika), pH adjusted to 7.0 with NaOH). Digestion was stopped by addition of DMEM (Life Technologies) with 10% fetal bovine serum (FBS; Life Technologies) and DNase I (Sigma), followed by 5 min centrifugation at 2000*g*. The supernatant was removed, and DMEM with 10% FBS and 3 μg/mL DNase I was added to the pellet, followed by resuspension of the cells and 3 min centrifugation at 2000*g*. Fresh DMEM with 10% FBS and DNase I was added to the cells. The cells were counted and 1 × 10^6^ cells/well were seeded out in a 12‐well tissue culture plate coated with poly‐L‐lysine (Sigma) and laminin (Invitrogen). After 1 day, half of the media was replaced with neurobasal A medium (Life Technologies) containing 2 mM GlutaMAX (Life Technologies), 2% B‐27 serum‐free supplement (Life Technologies), 0.2% primocin (Invitrogen), and 0.1% floxuridine and uridine (Sigma), in which the cells were maintained. The cells were kept in 50% conditioned media at 37°C and 5% CO_2_. BDNF treatment was initiated at day 8 in 50% conditioned media by adding hBDNF (Alomone Labs) to a final concentration of 54 ng/mL to each well at the indicated time points.

### 2.8 DNA purification and genotyping of Bdnf^+/−^ and Bdnf^+/+^ mice

DNA was purified from tail pieces or ear punches with Wizard Genomic DNA Purification Kit (Promega) according to the manufacturer's protocol. DNA concentrations were measured on a NanoDrop Lite Spectrophotometer (Thermo Scientific). PCR reactions were performed in 10% DMSO (Sigma), 0.5 mM dNTP (Invitrogen), 0.005% BSA, 25 U/mL Taq‐polymerase (Invitrogen), 10 μM forward and reverse primer and 10 ng DNA template. The PCR cycling conditions were as follows: 1 cycle of 3 min at 95°C, 5 min at 60°C, 40 cycles of 30 s at 95°C, 50 s at 60°C, 2 min at 65°C, 30 s at 4°C, and 1 cycle of 3 min at 95°C and 10 min at 65°C. DNA was mixed with 1× DNA loading buffer [0.25% bromophenol blue (Sigma), 0.25% xylene cyanal (Sigma), 30% glycerol (Sigma)], and run on a 2% agarose gel (Invitrogen) with 1:20,000 GelRed nucleic acid stain (VWR). PCR products were visualized with UV light. Forward primer used to detect BDNF was 5′‐AT AAA GAA GTA AAC GTC CAC‐3′, the reverse primer was 5′‐CCA GCA GAA AGA GTA GAG GAG‐3′. The forward primer used to recognize the knockout allele of BDNF was 5′‐CGG CGC CCA TGA AAG AAG TAA AC‐3′, and the reverse was 5′‐AAA GCG CAT GCT CCA GAC TGC CTT‐3′ (all from Sigma).

### 2.9 Cell extracts for Western blotting

Cells were lysed in TNE lysis buffer [10 mM Tris–HCl, 1 mM EDTA (pH 8.0; Sigma), 1% NP‐40 (Sigma) and 10% complete mini protease inhibitor cocktail (Roche) and 10% PhosSTOP phosphatase inhibitor cocktail (Roche)] on ice followed by sonication. Extracts were stored at −80°C. Bradford protein assay was used for protein concentration determination.

### 2.10 Western blotting

Fifteen to forty microgram whole cell extract (WCE) or tissue extract was boiled in SDS NuPage Loading Dye (Novus Life Technologies) before loading on a 7% TA polyacrylamide gel. The gel was run for 1 h 10 min at 150 V in 1× TA running buffer. The gel was rinsed in ddH_2_O before dry transfer at 20 V for 1 min, 23 V for 4 min and 25 V for 2 min using the iBlot2 blotting system (Life Technologies). After transfer, the membrane was rinsed in TBS‐T, blocked with 5% low fat skim milk‐TBS‐T, followed by incubation with primary antibody ON at 4°C. The primary antibodies used were: rabbit anti‐POLB (Abcam #ab175197) 1:2000, rabbit anti‐NEIL2 (Abcam #ab180576) 1:5000, rabbit anti‐APE1 (Thermo Scientific #PA5‐29157) 1:2000, rabbit anti‐phospho‐AKT (Ser473; Cell signaling #CST9271S) 1:1000, rabbit anti‐phospho‐CREB (Ser133; Cell signaling #CST9198S) 1:1000, mouse anti‐Actin (Sigma #A2228) 1:20,000, rabbit anti‐BDNF (Abcam #ab108319) 1:1000, and mouse anti‐GAPDH (Sigma #G8795) 1:10,000. After incubation with primary antibody the membrane was washed in TBS‐T, then incubated with secondary antibody for 1 h at RT, washed in TBS‐T and detected with ECL prime (Amersham). Secondary antibodies used were: anti‐rabbit IgG horseradish peroxidase‐linked (GE Healthcare #NA934) 1:5000 and anti‐mouse IgG horseradish peroxidase‐linked (GE Healthcare #NA931) 1:5000. Because of very similar size of proteins, membranes were, when necessary, stripped with Restore PLUS Western blot stripping buffer (Thermo Scientific) before re‐probing with antibodies against other antigens. Western blots were quantified by use of ImageJ software.

### 2.11 Cell and tissue extract for activity assays

Three to four million cells or 10 mg tissue were used per treatment or genotype. Cells were scraped off the tissue culture plates in cold PBS containing 1 mM PMSF and 1 mM DTT, then centrifuged at 2000*g* for 5 min at 4°C. The supernatant was removed, and the pellet frozen in dry ice‐ethanol slurry. Pellets were thawed on wet ice, and 150 μL Buffer I (10 mM Tris pH 7.8, 200 mM KCl) was added. Samples were sonicated, followed by addition of 150 μL Buffer II (10 mM Tris pH 7.8, 200 mM KCl, 2 mM EDTA, 40% glycerol, 0.2% NP‐40, 4 mM DTT, 1 mM PMSF, 20 μg leupeptin, 4 μg pepstatin). Samples were vortexed and then rotated for 2 h at 4°C. Then, samples were dialyzed for 1.5 h in Slide‐A‐Lyzer® MINI Dialysis Units (cut off 7000 MWCO; Thermo Scientific) against 1 L GDB (10% glycerol, 50 mM KCl, 25 mM Hepes‐KOH pH 7, 2 mM EDTA, 2 mM DTT). Concentrations were determined by Bradford protein assay. Tissue extract was used for both activity assays and Western blotting.

### 2.12 Incision assays

Incision activities were measured by incubating WCE with radioactively labeled oligomers containing enzyme‐specific targets as explained below. This is a reliable way of measuring changes in activity of specific DNA repair proteins, but we cannot exclude a minor contribution from backup enzymes. APE1 incision activity was determined by measuring the incision of a double‐stranded oligomer containing an AP site analog [Tetrahydrofuran (THF)], of which the vast majority of activity toward the lesion in WCE will be derived from APE1, or control oligomer without a lesion (Table S4). Twelve point five to four hundred nanogram WCE or 50–100 ng tissue extract, as indicated, was incubated with 0.5 nM THF oligomer under the final reaction conditions: 7.5% glycerol (Sigma), 100 mM KCl, 2 mM EDTA, 1 mM DTT, 2.5 mM MgCl_2_, 20 mM HEPES‐KOH (pH 7.0), for 15 min at 37°C. The reaction was terminated by addition of 20 μL FA loading buffer (80% formamide (Merck), 10 mM EDTA, 1 mg/mL xylene cyanol FF, 1 mg/mL bromophenol blue) and heated for 5 min at 90°C. NEIL incision activity was determined by measuring the incision of a double‐stranded substrate with an 11 base pair bubble in the middle of the sequence and containing a 5‐hydroxy uracil in the center of the bubble or control oligomer (Table S4). All three NEIL glycosylases can recognize this substrate, bind to and incise it, and therefore we call this activity *NEIL activity* onwards. One hundred to six hundred nanogram WCE or 3–4 μg tissue extract was used per reaction, containing 7.5% glycerol, 112.5 mM KCl, 3 mM EDTA, 1.5 mM DTT, 1 mM MgCl_2_, 18.75 mM Hepes KOH 7.0 and 0.5 nM oligomer. Samples were incubated at 37°C for 2 h, terminated by addition of 20 μL FA loading buffer with 130 mM NaOH, incubated for 15 min at 37°C, and then heated for 5 min at 95°C, as described in (Aamann et al., 2014). OGG1 incision activity was measured with a double‐stranded substrate with an 8‐oxoguanine or control oligomer without a lesion. Twenty five microgram tissue extract was used per reaction, containing 40 mM HEPES‐KOH (pH 7.0), 75 mM KCl, 1 mM DTT, 1.5 mM EDTA, 0.1 mg/mL BSA, 0.5 mM MgCl_2_, 7.5 mM dNTPs, and 5 nM oligomer. Samples were incubated at 37°C for 3 h. Hereafter, proteinase K was added to a final concentration of 200 ng/μL, SDS to a concentration of 0.5% and EDTA raised to 20 mM. Samples were incubated for 30 min at 55°C followed by addition of FA loading buffer and 2 min incubation at 80°C. Samples in all incision activity assays were separated on a 20% denaturing polyacrylamide gel, analyzed by phosphor imaging, and quantified by use of Image Quant software. Percentage incision was calculated as the amount of product relative to the total amount of product and non‐cleaved substrate.

### 2.13 Incorporation assay

Total BER synthesis including incorporation was determined by measuring the incorporation of [^32^P]‐dCTP into a hairpin looped oligomer containing a single uracil positioned in the stem of the hairpin Table S4. Five microgram WCE or 40 μg tissue extract was incubated with an uracil‐containing hairpin looped oligomer or control with 0.08 μCi/μl [^32^P]‐dCTP in reaction conditions (110 mM Hepes, 1.4 mM EDTA, 1 mM MgCl_2_, 0.25 mg/mL BSA, 70 mM KCl, 3.8 mM DTT, 0.04 mM phosphocreatine, 100 μg/mL phosphocreatine kinase, 2 mM ATP, 0.02 mM dNTPs) at 37°C for 3 h. DNA ligase (Invitrogen) was added to some samples (as indicated) to check if the incorporated products could be ligated, and reactions were terminated by 30 min treatment with 1.25 μg Proteinase K and 2.5 μL 10% SDS at 55°C. The DNA was isolated by phenol:chloroform extraction and precipitated in 96% EtOH in the presence of 167 mM ammonium acetate and 4 ng/μL glycogen ON at −20°C. The next day, the DNA was pelleted by centrifugation at 16,000*g*, washed in 80% EtOH, followed by drying of the DNA pellet, and resuspension in 20 μL FA loading buffer, before it was loaded on a 20% denaturing polyacrylamide gel. The incorporated radioactively labeled dCTP was visualized using phosphor imaging and quantified by use of image quant software.

### 2.14 Long‐range PCR for DNA damage analysis

Long‐range PCR was carried out essentially as previously described (Chakraborty et al., 2015; Furda et al., 2014) with some modifications. DNA was extracted from 15 mg hippocampal tissue using the QIAamp DNA mini kit (Qiagen) according to manufacturer's protocol. Quantification of DNA concentration was conducted with Quant‐iT PicoGreen dsDNA assay kit (Invitrogen). Preliminary tests indicated that the DNA extraction was gently enough for mtDNA to still be present in its supercoiled form. As this can affect the subsequent PCR (Chen et al., 2007), supercoiling was released prior to measurements by digestion with the methylation‐insensitive restriction enzyme BciVI (New England Biolabs), which cuts mtDNA outside regions amplified in the subsequent PCR reactions. DNA template was incubated with BciVI in 1× CutSmart buffer for 15 min at 37°C followed by heat inactivation for 20 min at 80°C. In order to induce strand breaks at sites of oxidized base lesions, the DNA template was incubated with Fpg enzyme (New England Biolabs; 15 U/mL for mtDNA and 50 U/mL for nuclear DNA) for 30 min at 37°C in a buffer containing 20 mM Tris–HCl pH 8, 0.5 mM EDTA, 50 mM NaCl, 200 μg/mL purified BSA and 50% glycerol followed by heat inactivation for 10 min at 60°C. Long‐range PCR was carried out for a 10 kb region of the mtDNA and 7.2 kb region of the NeuroD gene in the nuclear genome using LongAmp Taq DNA polymerase (New England Biolabs). Long PCR fragments were normalized to small PCR fragments for which amplification is assumed to be independent of DNA damage. The 10 kb mtDNA fragment was normalized to a 117 bp mtDNA fragment. The 7.2 kb NeuroD fragment was normalized to a 282 bp NeuroD fragment. Primers are listed in Table S5. Small PCR fragments were amplified with Taq DNA polymerase (New England Biolabs). Cycle number and DNA concentration was optimized for each PCR reaction to ensure measurements within the linear range of the reaction. In all cases, a 50% control sample containing half the amount of DNA was run. PCR conditions are displayed in Table S5. Amplified PCR fragments were visualized on agarose gels (Amersham Imager 600, GE Healthcare) and quantified using ImageQuant TL software. Lesion frequency per 10 kb was calculated as described in Ayala‐Torres et al. (2000).

### 2.15 Statistical analysis

Comparisons of groups were performed by Student's *t* test or one‐way ANOVA (Dunnett's post hoc test) with equal or unequal variance (Welch's correction for unequal variance) in Prism (v. 7.04). Data are displayed as mean and standard error of mean (SEM). Correlation analyses were conducted in R (v. 4.2.0). Spearman's rank correlation coefficient or partial Spearman's correlation coefficient adjusting for age was computed using the PResiduals package (Liu, Li, et al., 2018). *p*‐values were corrected for multiple testing by the Benjamini–Hochberg procedure. *p ≤ 0.05; **p ≤ 0.01; ***p ≤ 0.001, ****p ≤ 0.0001.

## 3 RESULTS

### 3.1 BER expression patterns in the aged human brain

**FIGURE 1:**
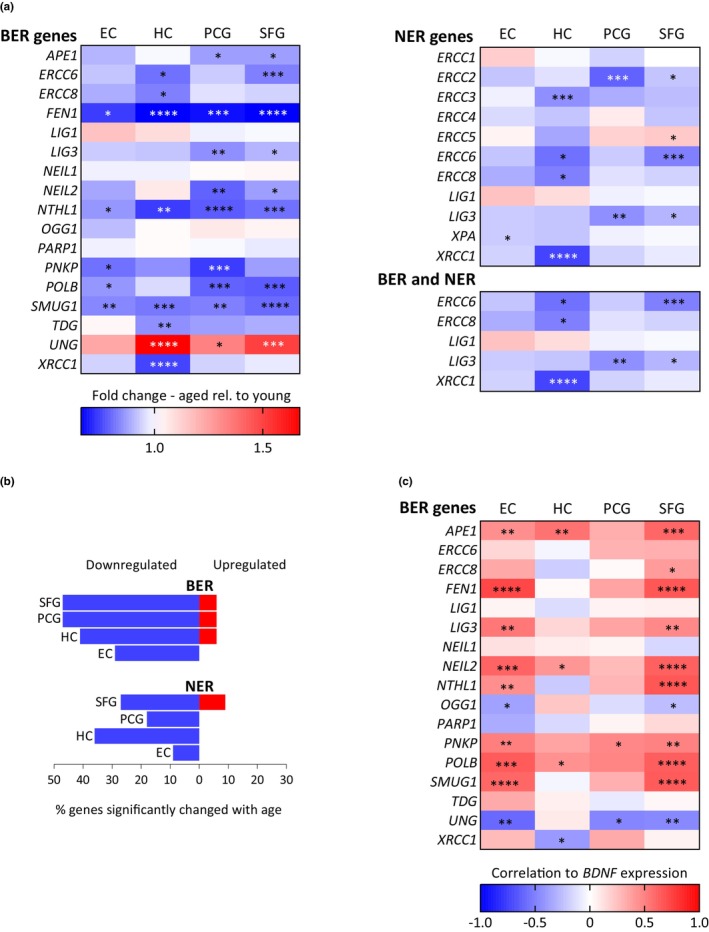
Transcriptional changes in DNA repair genes during aging and with *BDNF* expression in four human brain regions (a) Fold change in BER‐ and NER‐related gene expression in aged individuals (age 69–99 years, *N* = 33) compared to young individuals (age 20–59 years, *N* = 22) in four brain regions. For clarity, genes involved in both BER and NER are also presented in a separate panel. Blue: downregulated expression. Red: upregulated expression. (b) Percentage of BER and NER genes significantly up‐ or downregulated in the four brain regions in aged individuals (age 69–99 years, *N* = 33) compared to young individuals (age 20–59 years, *N* = 22). Genes involved in both pathways are included in both the BER and NER analysis. Changes were considered significant at *p* < 0.05. (c) Correlation between expression of *BDNF* and BER genes in four different brain regions from individuals aged 20–99 years (*N* = 57). Partial Spearman's rank correlation coefficient adjusted for age was computed. Benjamini‐Hochberg correction was performed for multiple testing. EC, Entorhinal cortex; HC, hippocampus; PCG, postcentral gyrus; SFG, superior frontal gyrus. *p ≤ 0.05; **p ≤ 0.01; ***p ≤ 0.001; ****p ≤ 0.0001.

In order to investigate whether the expression of genes involved in the BER pathway changes as the human brain ages, the expression profile of 17 core genes for the BER pathway and its sub‐pathways were analyzed in post‐mortem human brain samples from 22 young (age 20–59 years, mean age 35.4 years) and 33 aged (age 69–99 years, non‐demented, mean age 83.2 years) individuals (Table S1). To determine more specifically whether any potential changes were region‐specific, we assessed the gene expression profiles in the HC and three different cortical brain regions, the entorhinal cortex (EC), superior frontal gyrus (SFG), and postcentral gyrus (PCG). EC, HC, and SFG are known to undergo functional decline with aging and age‐associated neurodegeneration including accumulation of pathology, whereas PCG is normally relatively unaffected (Braak & Braak, 1991). Interestingly, the expression of a large fraction of the BER associated genes was downregulated with age in all four regions examined when comparing the aged group to the young group (Figure 1a; Table S2). Notably, the expression of the DNA glycosylase *NTHL1* involved in repair of oxidized pyrimidines, *FEN1*, the central protein in long‐patch BER, and the uracil DNA glycosylase *SMUG1* were significantly downregulated in all four regions (Figure 1a). In addition, the expression of *POLB*, the key polymerase in BER, was significantly downregulated in EC, SFG, and PCG (Figure 1a). Several other BER genes were also significantly downregulated with age in one or more of the brain regions including *APE1*, *ERCC6*, *ERCC8*, *LIG3*, *NEIL2*, *PNKP*, *TDG*, and *XRCC1* (Figure 1a). Approx. 50% of the BER genes examined were significantly downregulated in SFG and PCG, and around 30% and 40% were significantly downregulated in EC and HC, respectively (Figure 1b). This is in accordance with the global gene expression profile where most age‐associated changes occur in SFG and PCG and less in EC and HC (Berchtold et al., 2008). Moreover, besides the significantly downregulated genes, the majority of the remaining BER genes analyzed here showed a tendency toward downregulation in most regions. The uracil DNA glycosylase *UNG* was as the only BER gene significantly upregulated with approx. 1.5‐fold higher expression in aged individuals compared to young in HC, PCG and SFG (Figure 1a). Correlation analysis of expression of BER genes and age revealed a significant negative correlation for *APE1*, *ERCC6*, *FEN1*, *LIG3*, *NEIL2*, *NTHL1*, *PNPK*, *POLB*, *SMUG1*, *TDG*, and *XRCC1* in at least one of the analyzed brain regions (Figures S1 and S2; Table S6). Moreover, the majority of the remaining downregulated BER genes showed a strong trend toward a negative correlation between expression and age. This suggests that most of the BER genes downregulated with age display a progressive change in expression during adult life rather than a decline after reaching a specific age.

To test whether it is especially the BER pathway that is affected in the aging brain, the gene expression profile of 12 core genes in the nucleotide excision repair (NER) pathway were also analyzed (Figure 1a; Table S2). The NER pathway mainly removes bulky DNA lesions introduced as a result of the attack by primarily exogenous DNA damaging agents. However, some gene products participate in both BER and NER, and the NER pathway has also been suggested to play a role in BER by stimulating and cooperating with the BER pathway in removal of oxidative DNA damage (Kumar et al., 2020). Compared to BER, a lower percentage of NER genes were downregulated in aged individuals compared to young (Figure 1b). The expression of four out of 11 (36%) NER genes (*ERCC2*, *ERCC6*, *LIG3*, and *XRCC1*) significantly negatively correlated with age in one or two brain regions. It is worth noting that out of the significantly changed genes both *ERCC6*, *LIG3*, and *XRCC1* are also involved in BER. For BER‐related genes, 11 out of 17 (65%) significantly negatively correlate with age in one or more brain regions (Figures S1 and S2; Table S6). Consequently, age‐associated changes occur for genes in both pathways in the human brain, however the higher number of genes downregulated in BER compared to NER in all brain regions examined, indicates that the BER pathway could be a particularly vulnerable DNA repair mechanism in the aging brain.

### 3.2 BER expression patterns correlate with BDNF expression in the human brain

The observed parallel age‐associated decline in many of the BER genes in the human brain suggests that a common regulator may exist that controls BER in a coordinated manner. Like BER, *BDNF* expression was downregulated in the aging human brain. Specifically, *BDNF* expression was significantly downregulated by around 60% in EC, SFG, and PCG, and by around 10% in the HC for aged individuals compared to young individuals (Table S2). This is consistent with previous reports of age‐related downregulation in BDNF levels in the brain and in agreement with a lower or differential impact of age on HC in regard to BDNF levels compared to cortical brain regions (Hattiangady et al., 2005; Katoh‐Semba et al., 1998; Oh et al., 2016; Webster et al., 2006). To investigate if BDNF could be a common regulator of the expression of the BER genes in the human brain we evaluated the correlation between expression of *BDNF* and each of the core BER genes in EC, SFG, PCG, and HC. Interestingly, the correlation analysis revealed a positive correlation between *BDNF* expression and many of the BER genes (Figure 1c). Excluding age as a co‐variate caused a slight reduction in the strength of the correlations (Figure S3). However, the directionality and significance of the correlations were generally maintained except for PCG in which many correlations disappeared after adjustment for age. Overall, the BER genes affected the most by aging also exhibited the strongest correlation to BDNF, and the strongest correlations were present in EC and SFG, whereas HC and PCG on average displayed weaker correlations. *POLB*, *APE1*, *NEIL2*, and *PNKP* expression were significantly, positively correlated to *BDNF* expression in three out of four brain regions, and *FEN1*, *LIG3*, *NTHL1*, and *SMUG1* in EC and SFG (Figure 1c). On the other hand, *UNG* exhibited a moderate to strong negative correlation in EC, PCG, and SFG and *XRCC1* and *OGG1* a weak to moderate negative correlation in one or two regions, respectively. We also examined the correlation between NER expression and BDNF expression in the four human brain regions. As seen in Figures S4 and S5 and Table S7, *ERCC2*, *ERCC8*, and *LIG3* showed a significant positive correlation with *BDNF* expression in at least one brain region, whereas *ERCC5*, *XPA*, and *XRCC1* showed a significant negative correlation with *BDNF* expression. The percentage of genes displaying a positive correlation to BDNF was higher in all brain regions examined for BER‐ than NER‐related genes (Figure S5B) indicating a stronger link between BDNF and BER than BDNF and NER. Collectively, these results suggest a role of BDNF in regulating expression of many BER genes in the human brain and that this regulation is region‐specific and extends beyond age‐dependent effects.

### 3.3 CREB binds in silico predicted CRE sites in many BER promoters

Since the correlative analysis indicates that BDNF could be a regulator of BER genes, we evaluated whether CREB could act as a possible downstream transcriptional regulator in this context. CREB regulates gene expression by binding to cAMP‐responsive elements (CRE) occurring as either full (TGACGTCA) or half sites (CGTCA/TGACG). It has previously been demonstrated that the promoters of *POLB* and *APE1* contain putative CRE sites, and that expression of *POLB* and *APE1* depends on these sites and on activated CREB (Grösch & Kaina, 1999; Narayan et al., 1995, 1996; Yang et al., 2010, 2014).

**FIGURE 2:**
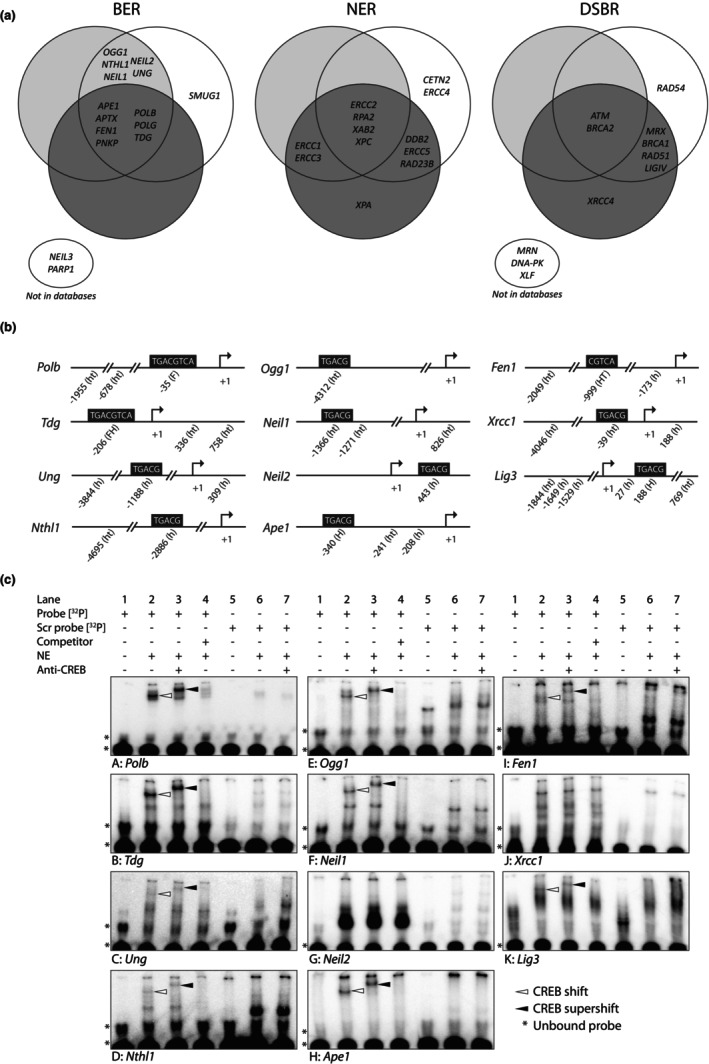
CREB binds to the majority of in silico predicted CRE sites in promoters of BER genes (a) CRE site prediction in BER, NER, and DSBR genes in the human genome. Two different databases were used for CRE site prediction: Champion ChIP database (Qiagen; light gray) and CREB target database (white). CREB target database was also used for search in the ChIP‐on‐chip database (dark gray). Lower small circles show genes not found in the databases. The genes shared between pathways are not shown here. (b) Position of predicted CRE sites in the promoter of selected BER genes in the mouse genome based on CREB target database. F/f: full site. H/h: half site. Uppercase letter: conserved CRE (human‐mouse‐rat). Lowercase letter: CRE not conserved (human‐mouse‐rat). FH: full site CRE in species studied but only half site in other species. T/t: presence of TATA box less than 300 bp downstream of CRE site. Number marks position relative to TSS (mm3 genome). CRE site in black box: site investigated in EMSA. (c) EMSA with mouse brain nuclear extracts (NE). Assays conducted with ^32^P‐labeled probes corresponding to potential CRE sites based on in silico predictions and immediate up‐ and downstream flanking sequence of eleven BER promoters (a–h). Lane 1: ^32^P‐labeled probe without NE. Lane 2: ^32^P‐labeled probe + NE. White arrowhead indicates CREB shift. Lane 3: ^32^P‐labeled probe + NE + anti‐CREB antibody. Black arrowhead indicates CREB super shift. Lane 4: ^32^P‐labeled probe + NE + competitor (unlabeled POLB probe in molar excess). Lane 5–7: identical to lane 1–3 except ^32^P‐labeled scrambled (Scr) probe (CRE site has been scrambled while flanking sequence was unchanged). * marks unbound probe.

By in silico predictions based on two databases and a ChIP‐on‐chip database search (Zhang et al., 2005), we verified those findings and in addition we found that promoters of many additional human core BER genes contain potential CREB‐binding sites (Figure 2a). Moreover, the majority of the BER promoters contained multiple CREB‐binding sites, where many were located close to a TATA box and several sites were conserved, increasing the likelihood of being functionally relevant sites (Conkright et al., 2003; Table S8). A similar in silico prediction was performed on genes associated with two other DNA repair pathways, NER and double‐strand break repair (DSBR), since CREB is known to target numerous promoters in the genome. Interestingly, out of the three DNA repair pathways, the promoters of genes encoding proteins associated with BER displayed the most consistent pattern across databases indicating that CREB may be particularly engaged in the regulation of this pathway (Figure 2a).

To study whether CREB is capable of binding to the predicted sites, we performed electrophoretic mobility shift assays (EMSAs) with nuclear protein extracts from mouse brain and DNA probes corresponding to selected in silico predicted CREB‐binding sites and flanking promoter regions of BER related genes in the mouse genome. Like human BER related genes, many of the promoters of BER associated genes in the mouse genome contain in silico predicted sites (Table S8). For eleven of the core BER genes we selected one of the most likely functional CRE sites in their promoter based on conservation, position relative to transcription start site (TSS) and the presence of TATA boxes nearby (Figure 2b). We designed 50 bp long DNA probes containing the CRE site under investigation, centrally positioned in the probe and flanked by immediate up‐ and downstream promoter sequences of the particular promoter investigated (Table S3). As expected, incubating a DNA probe containing the full CRE site and flanking region of the *Polb* promoter with nuclear extract led to a band shift (Figure 2c, panel A, lane 2, white arrowhead) and CREB was identified as the bound protein in the shifted complex by addition of anti‐CREB antibody, which resulted in a super shift (Figure 2c, panel A, lane 3, black arrowhead). Notably, CREB bound to nine out of eleven (82%) predicted CRE sites examined here (Figure 2c). In all instances band shifts due to CREB binding disappeared when the sequence of the putative CRE sites were scrambled (Figure 2c, lane 5–7) and in competitive assays with molar excess of unlabeled *Polb* promoter probe, respectively (Figure 2c, lane 4). These important controls confirm that CREB binds specifically to the CRE sites under investigation. Accordingly, our results suggest that CREB has the potential to be involved in a widespread control of the BER pathway by regulating the expression of several BER genes exhibiting different types of enzymatic activities and functioning in different steps of BER: *Ogg1*, *Tdg*, *Ung*, *Nthl1*, *Ape1*, *Fen1*, *Polb*, *Lig3*, but not *Neil2* and *Xrcc1* (Figure 2c).

Besides the sequence flanking the CRE site, CREB binding to a CRE site can be influenced by methylation and the DNA methylome is known to be affected by aging in the brain (Prasad & Jho, 2019). Methylation at the central CpG sequence in a full CRE site has been shown to reduce the affinity of CREB for its binding site in a manner independent of which strand the methylation is situated on (Kitsera et al., 2017). Indeed, we observed that upon methylation of the half CRE site in the *Neil1* and *Ogg1* promoter, respectively, CREB binding present in the unmethylated state is completely abolished upon methylation (Figure S6). To elucidate whether the predicted CRE sites in the BER promoters may be affected by methylation at old age, DNA was isolated from the brain of middle‐aged to old mice (11–28 months) and the methylation status examined at selected promoter regions of the BER genes. The CRE site in the promoter of *Ape1* and *Polb* were not methylated in any of the mice, suggesting that observed age‐associated changes in *Ape1* and *Polb* expression in the brain are not due to methylation of the CRE site (Figure S7). On the other hand, the three other BER genes investigated, *Neil1*, *Ung*, and *Ogg1*, displayed a medium to high level of methylation at the CpG dinucleotide in the CRE site investigated in both middle‐aged and old mice (Figure S7).

### 3.4 BDNF treatment stimulates BER in primary hippocampal neurons

Besides the extend of CREB binding to CRE sites, induction of gene expression by CREB depends on its activation as occurs via, for example, BDNF signaling. Our correlative BDNF findings in the human brain (Figure 1) and our CREB‐binding studies (Figure 2) suggest that BDNF possibly via CREB could be a major regulator of BER gene transcription. Therefore, we wanted to explore whether there is a causative connection between BDNF and BER. To evaluate this, we treated primary hippocampal neurons from mice and rats, respectively, with BDNF for various timepoints and assessed the protein expression and activity of a selected subset of BER enzymes.

**FIGURE 3:**
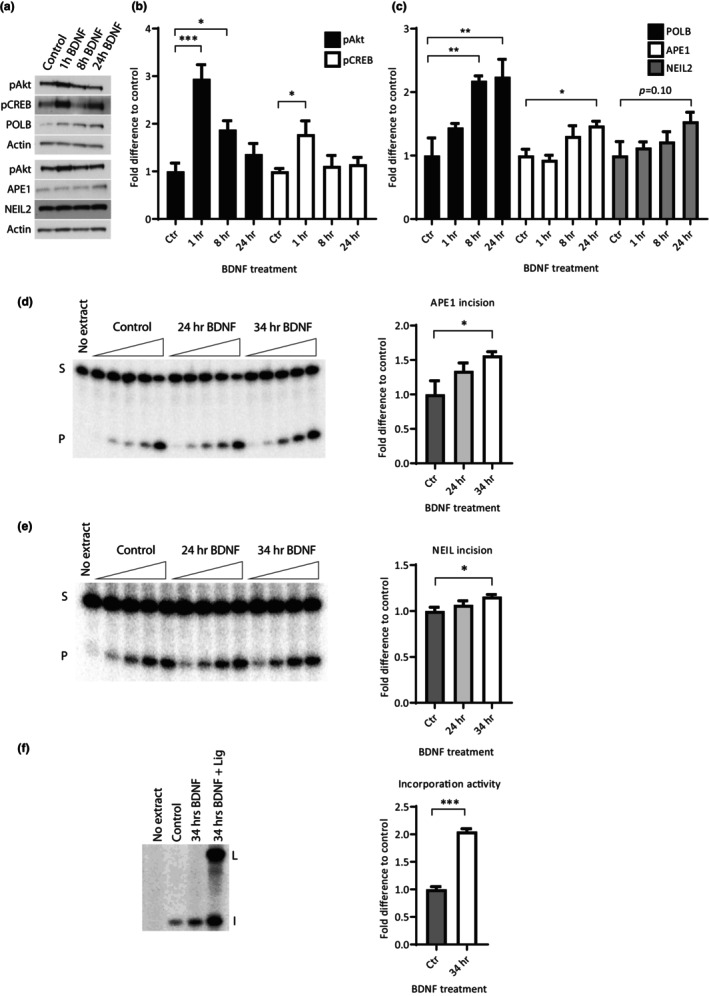
Activation of intracellular signaling and increased BER protein expression and activity after BDNF treatment in primary mouse hippocampal neurons. Primary neurons were treated with 54 ng/mL BDNF for indicated time periods or control without BDNF treatment. Activation of intracellular signaling and BER protein expression were evaluated by immunoblotting. (a) Representative immunoblots. Proteins probed for are shown on the left. (b) Quantification of pAkt and pCREB levels. (c) Quantification of BER protein expression. Values are relative to Actin level. DNA repair activity of indicated BER enzymes toward DNA oligomers containing specific lesions was evaluated in neuronal extracts. Left panels shows a representative gel and right panels quantification from three independent cultures. (d) APE1 incision activity with increasing amount of extract incubated with a 5′‐^32^P‐labeled double‐stranded oligomer containing a THF lesion (AP‐site analog). (e) NEIL incision activity with increasing amount of extract incubated with a 5′‐^32^P‐labeled partially double‐stranded oligomer with an internal 11 nt bubble containing a 5‐hydroxyuracil lesion. (d + e) Values are the average of measurements conducted at increasing amounts of protein extract within the linear range of the assay. S: Substrate/non‐cleaved oligomer. P: Product/cleaved oligomer. (f) Incorporation activity. Neuronal extracts were incubated with a DNA hairpin containing an uracil lesion and ^32^P‐dCTP. + Lig: Addition of T4 DNA ligase. I: Incorporation products. L: Ligation products. All values are fold difference compared to control and are mean and SEM (*n* = 3 independent cultures). *p ≤ 0.05; **p ≤ 0.01; ***p ≤ 0.001.

As expected, treatment of mouse hippocampal neuronal cultures with BDNF for 1 h activated intracellular signaling. In particular, a three‐fold increase in phosphorylation of the kinase Akt and almost two‐fold increase in phosphorylation of CREB was observed in WCE (Figure 3a,b). APE1 protein level was significantly increased after 24 h BDNF treatment (1.5‐fold, Figure 3a,c) corroborating with what was previously reported for rat cortical neurons (Yang et al., 2014). Interestingly, BDNF treatment from 1 to 24 h led to increased protein level of POLB, which was upregulated already after 1 h BDNF treatment (1.5‐fold), continued to increase to more than two‐fold after 8 h, and remained at this elevated level during the 24 h evaluated (Figure 3a,c). NEIL2 displayed a tendency toward increased protein expression (1.5‐fold, *p*‐value = 0.10) after BDNF treatment (Figure 3a,c). In addition, our preliminary data confirm a similar BDNF stimulated upregulation of pAkt, APE1, NEIL2, and FEN1 levels in rat hippocampal neurons (Figure S8). Unfortunately, availability of functional antibodies recognizing BER proteins with high specificity limited our analysis of additional core BER proteins.

To verify that the observed increased protein expression also leads to increased DNA repair capacity in the mouse hippocampal neuronal cultures, we measured the activity of central BER proteins by in vitro DNA repair assays. By incubating neuronal extracts with a DNA oligomer containing a single specific lesion at a specific site, the incision and/or incorporation activity of the neuronal extract was examined. Depending on the exact lesion introduced in the DNA oligomer, specific repair enzyme activities can be analyzed (see Material and Methods for details) with only minor contributions from backup enzymes (Alexeeva et al., 2021; Gros et al., 2004; Li et al., 2014; Muftuoglu et al., 2009). BDNF treatment of mouse hippocampal neurons for 24 and 34 h, respectively, increased APE1 incision activity and resulted in a significant 1.5‐fold increase after 34 h (Figure 3d). This is similar to the extent of protein upregulation, suggesting that the increase in APE1 activity can largely be explained by increased protein expression. Additionally, the NEIL incision activity corresponding to the activities of NEIL1‐3 (Dou et al., 2003; Liu et al., 2010), displayed a significant 15% increase after 34 h of BDNF treatment (Figure 3e) in agreement with an increase in NEIL2 protein expression. Interestingly, we found a significant two‐fold increase in incorporation activity after 34 h BDNF treatment (Figure 3f). Incorporation activity is a measure of the BER pathway as a whole from recognition of damaged base in the DNA to the incorporation of a new nucleotide by POLB. Incorporation products were checked for ligation ability by addition of DNA ligase to a subset of the samples. Collectively, these results indicate that BDNF plays a direct role in regulating BER that extends beyond regulation of APE1 and suggest that POLB and NEIL2 and possibly other BER enzymes are regulated by BDNF as well.

### 3.5 DNA repair capacity in Bdnf heterozygous mice

**FIGURE 4:**
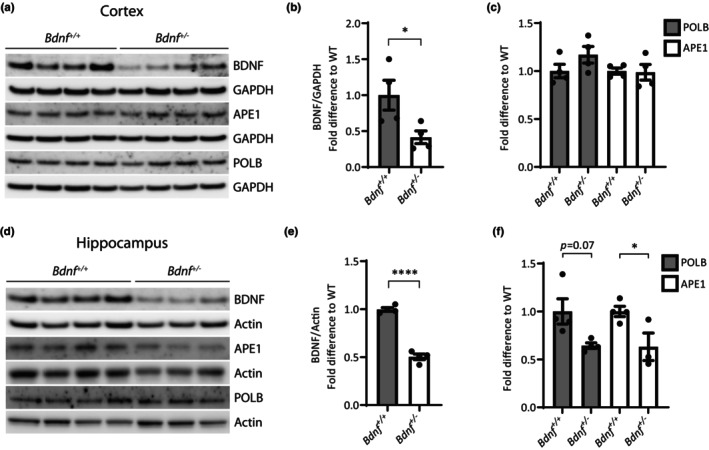
BER protein expression is reduced in HC of *Bdnf* ^+/−^ mice BDNF and BER protein expression measured by immunoblotting in *Bdnf* ^+/+^ and *Bdnf* ^+/−^ mice. (a–c) Cortex, *n* = 4 mice/genotype. (d–f) HC, *n* = 4 *Bdnf* ^+/+^ and 3 *Bdnf* ^+/−^ mice. Left panels shows representative immunoblots. Values are relative to Actin or GAPDH level. All values are fold difference compared to *Bdnf* ^+/+^ mice and values are mean and SEM. *p ≤ 0.05; ****p ≤ 0.0001.

In light of the identified regulatory role of BDNF, we investigated the consequence of reduced BDNF levels in vivo, as observed in the aging brain. In order to better resemble the reduction in BDNF seen in normal aging and confirmed in our study population (Table S2), we utilized mice heterozygous for *Bdnf* (*Bdnf* ^+/−^ males, 4‐months old) that displayed a 50% reduction in BDNF protein expression in the brain compared to wildtype mice (Figure 4b+e). Moreover, BDNF knockout mice are not viable (Ernfors et al., 1994; Linnarsson et al., 1997). We examined the level of selected BER proteins in extracts made from HC and cortex, respectively. In the HC, we observed a reduction in POLB and APE1 protein level by approx. 35% (Figure 4f) in *Bdnf* ^*+/−*^ mice, whereas the POLB and APE1 protein level was not affected by reduced BDNF in cortex (Figure 4c) suggesting brain regional differences in the response to decreased BDNF.

**FIGURE 5:**
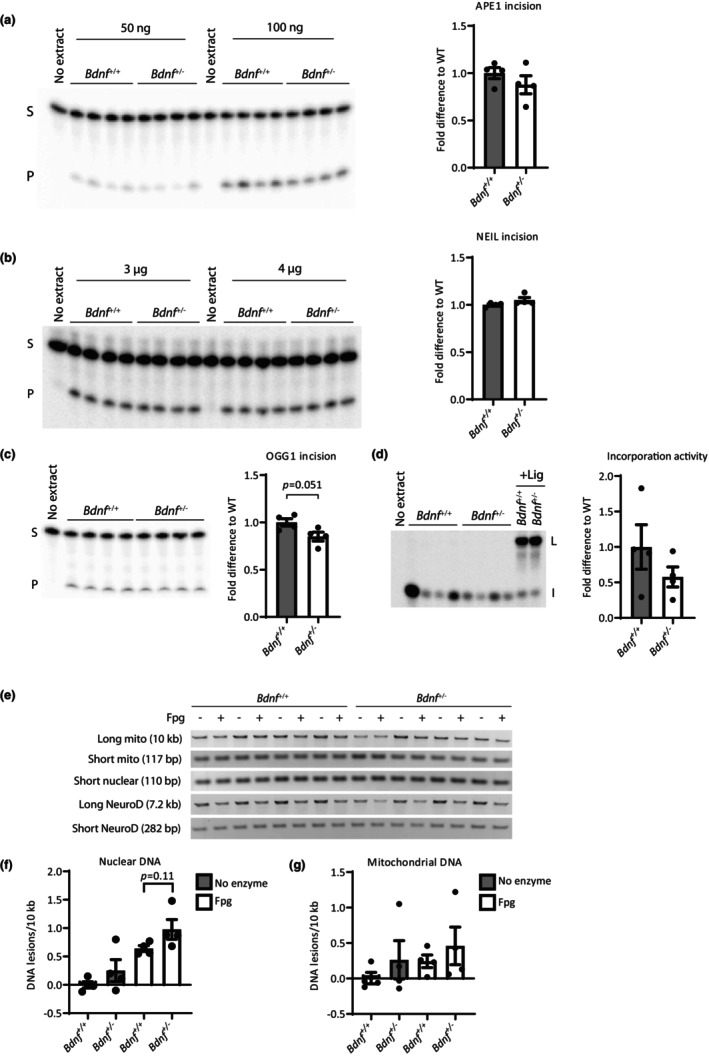
BER activities and DNA damage in HC of *Bdnf* ^+/−^ mice (a–c) DNA repair activity of indicated BER enzymes in HC of *Bdnf* ^+/+^ and *Bdnf* ^+/−^ mice. Left panels shows representative gels. S: substrate or non‐cleaved oligomer. P: product or cleaved oligomer. (a) APE1 incision activity. Hippocampal extract incubated with 5′‐^32^P‐labeled double‐stranded oligomer containing a THF lesion (AP‐site analog) (b) NEIL incision activity. Hippocampal extract incubated with a 5′‐^32^P‐labeled partially double‐stranded oligomer with an internal 11 nt bubble containing a 5‐hydroxyuracil lesion. (a + b) Values are the average of measurements conducted at increasing amounts of protein extract. (c) OGG1 incision activity. Hippocampal extract incubated with a 5′‐^32^P‐labeled double‐stranded oligomer containing an 8oxoG lesion. (d) Incorporation activity. Hippocampal extracts were incubated with a DNA hairpin containing an uracil lesion and ^32^P‐dCTP. + Lig: Addition of T4 DNA ligase. I: incorporation products. L: ligation products. All values are fold difference compared to control and are mean and SEM. The level of DNA damage was evaluated in the HC of *Bdnf* ^+/+^ and *Bdnf* ^+/−^ mice by long‐range PCR in the nuclear and mitochondrial genome. DNA was digested with Fpg to reveal oxidized base lesions in the analysis. (e) Representative gels. (f) DNA damage in nuclear DNA was assessed in a 7.2 kb region of the NeuroD gene and normalized to a 282 bp fragment of the NeuroD gene. (g) DNA damage was assessed in a 10 kb region of the mtDNA and normalized to a 117 bp fragment of the mtDNA to account for any differences in mtDNA copy number. (f + g) Values are expressed as lesion frequency/10 kb of DNA. *N* = 4 mice/genotype. Values are fold difference compared to *Bdnf* ^+/+^ mice and are mean and SEM.

POLB heterozygosity has previously been shown to affect the level of DNA damage, mitochondrial function, and olfactory function in the 3xTgAD mouse model (Misiak et al., 2017; Sykora et al., 2015). To test whether reduction in APE1 and POLB expression in the HC of *Bdnf* ^+/−^ mice affect the BER repair activity, we used in vitro DNA repair activity assays as described above with extracts from HC. The incision activity of APE1 was reduced by 15% in the HC of the heterozygotic mice although not statistically significant (Figure 5a). NEIL incision was unchanged (Figure 5b). On the other hand, the incision activity of OGG1, the enzyme responsible for repair of one of the most common oxidative base lesions, 8oxoG, also showed a tendency toward 15% reduction in activity (Figure 5c, *p*‐value = 0.051). Finally, we measured incorporation activity of the hippocampal extracts. There was a tendency toward decreased incorporation activity in the *Bdnf* ^*+/−*^ mice compared with *Bdnf* ^*+/+*^; however, it did not reach statistical significance due to a high variation observed within the group of *Bdnf* ^*+/+*^ mice (Figure 5d).

To examine the level of DNA damage in the HC of the *Bdnf* ^+/−^ mice, the relative level of endogenous DNA damage in the nuclear and mitochondrial genome was evaluated by long‐range PCR. Increased DNA damage will be observed as a decrease in the PCR amplification due to blockage or stalling of the DNA polymerase when encountering damage during the PCR reaction. To evaluate oxidized base lesions, the DNA was treated with Fpg which converts these lesions into single strand breaks that efficiently block the DNA polymerase. In a 7.2 kb region of the NeuroD gene in the nuclear genome the *Bdnf* ^+/−^ mice displayed a slight, but non‐significant increase in the frequency of DNA lesions compared to WT (Figure 5e,f, no enzyme). For the Fpg treated samples, there was a tendency toward an increase in DNA lesions in the nuclear genome of *Bdnf* ^+/−^ mice compared to the WT mice (*p*‐value = 0.11; Figure 5e,f, Fpg). A similar trend was observed in a 10 kb region of the mitochondrial genome of *Bdnf* ^+/−^ mice exhibiting slightly elevated, but non‐significant, level of DNA lesions (Figure 5e,g).

## 4 DISCUSSION

The BER pathway, a critical DNA repair mechanism in the brain, represents a promising therapeutic target for intervention in age‐associated neurodegenerative diseases. However, despite extensive clarification of the pathway mechanistics, insights into age‐related changes in BER as well as regulatory mechanisms controlling BER in the brain are very limited. Furthermore, most studies have been conducted in rodents although age‐associated changes in gene expression in the brain are not well‐conserved between species (Loerch et al., 2008).

Here we show for the first time a collective gene expression landscape of the core BER and NER genes during human brain aging and find that the expression of especially core BER genes are downregulated with age across brain regions. We explore the regulatory effect of the neurotrophic factor BDNF on BER and NER gene expression. We detect a close association between the expression of multiple BER genes and *BDNF* expression in the human brain, which suggests BDNF as a major regulator of BER genes. This close association was largely unaffected by adjustment for age as a co‐variate, indicating that changes in *BDNF* expression are directly influencing age‐related changes in BER expression. Our analysis finds a higher percentage of BER genes than NER genes with a positive correlation to *BDNF* expression, and additionally, the correlation between BER and *BDNF* expression is in general stronger compared with the correlation between NER and *BDNF* expression. Our results points toward a more central role of the BDNF‐CREB axis in regulating BER gene expression than NER in the human brain but does not exclude the possibility that BDNF also plays a role in regulating NER.

Based on the association between BER and BDNF expression in the human brain, we test whether there is a causal link between BDNF signaling and BER. First, we perform an extensive search for the occurrence of potential CRE sites by in silico predictions and in vitro CREB‐binding studies for the majority of BER promoters. These results suggest that CREB, a major downstream effector of BDNF signaling, might exert transcriptional control over many of the BER genes. Secondly, we investigate the effect of BDNF stimulation on the expression and activity of a selected number of BER genes in mouse hippocampal neurons. These results pinpoint BDNF as a notable activator of several key BER genes extending beyond its previously described role in regulating APE1 in rat cortical neurons (Yang et al., 2014). Notably, the results in the mouse hippocampal neurons are consistent with the associations found in human HC for *APE1*, *POLB*, and *NEIL2*. Collectively, this indicates BDNF as a common regulator of BER in the brain by coordinating a balanced adjustment of enzyme levels in the different steps of BER and thereby avoiding accumulation of potentially mutagenic repair intermediates.

There are several reports of accumulation of oxidative DNA damage during human brain aging. The level of 8oxoG, a widely used marker of oxidative DNA damage, has been shown to progressively increase with age in nuclear and mitochondrial DNA in the human brain (Mecocci et al., 1993). Importantly, Yankner and colleagues have shown that oxidative DNA damage accumulates in the promoter and exons of genes in the aged human cortex. Moreover, they demonstrate that accelerated accumulation contributes to reduced gene expression, which likely plays a key role in the aging process of the human brain (Lu et al., 2004). Build‐up of oxidative DNA lesions in the genome is a consequence of an imbalance between ROS production, elimination of ROS by the antioxidant defense system, and repair of ROS‐induced DNA lesions by BER. Based on our results, it is likely that a major component of this imbalance is the progressive downregulation of BER genes during aging that takes place across different cortical regions in the human brain.

Only one of the BER genes, *UNG*, was upregulated in the brain during aging in our cohort, validating previous findings in the aging human frontal cortex (Lu et al., 2004). The fact that the *UNG* gene displays an aberrant age‐associated change in expression compared with the other BER genes suggests that the underlying reason may be related to non‐canonical roles of *UNG* outside the BER pathway such as antibody class switching (Stratigopoulou et al., 2020; Yousif et al., 2014). It is also worth noting that several miRNAs regulate UNG in various cell types (Hegre et al., 2013) and that many miRNAs are differentially expressed with aging in the brain (Danka Mohammed et al., 2017).

In this study, we observe for the first time a direct link between BDNF and POLB in the brain. Interestingly, *POLB* expression displays one of the strongest associations to BDNF across brain regions in the human brain and the highest increase in protein expression upon BDNF stimulation in mouse hippocampal neurons. It is well‐established that the *POLB* gene contains a CRE site in its core promoter essential for full promoter activity (Englander & Wilson, 1990; Widen et al., 1988; Widen & Wilson, 1991; Yamaguchi et al., 1994). Moreover, previous studies have shown that POLB expression is stimulated in a CREB‐dependent manner in response to DNA damaging agents in mammalian cell lines (He et al., 2003; Kedar et al., 1991; Narayan et al., 1995, 1996; Wang et al., 2001; Zhao et al., 2012). However, in primary cortical neurons many previously investigated stimuli, which alter APE1 in a CREB‐mediated way, do not appear to affect POLB expression notably. This includes activation of both the glucagon‐like peptide‐1 (GLP‐1) and glutamate receptor (Yang et al., 2010, 2016), respectively, as well as TrkB activation by BDNF (Yang et al., 2014). On the other hand, POLB expression is upregulated in response to ischemic injury in cerebral cortex whereas APE1 expression is not altered (Lan et al., 2003; Li et al., 2007). Although in this case the underlying regulatory mechanism was not investigated, it is possible that the BDNF‐CREB axis is involved since BDNF plays a vital neuroprotective role in ischemia (Chen et al., 2013). Bohr and Mattson's groups did not observe a BDNF‐induced increase in POLB expression in cortical neurons (Yang et al., 2014), whereas we find a large upregulation of POLB upon BDNF treatment of hippocampal neurons. Consistent with this divergent data in cortical and hippocampal neurons, several studies have demonstrated tissue‐ and cell‐type‐specific CREB target profiles although CREB is ubiquitously expressed. For example, well‐characterized CRE sites have been shown to display large differences in CREB occupancy between PC12 cells, H4IIE rat hepatoma cells, and cortical neurons (Cha‐Molstad et al., 2004). Moreover, a large‐scale study of >860 CREB‐binding sites in the rat brain demonstrated substantial regional differences in CREB occupancy between frontal cortex, HC, and striatum. In addition, it was shown that CREB stimulation in response to electroconvulsive seizure increased CREB occupancy only at a selected subset of CREB targets (Tanis et al., 2008). Several layers of regulation likely contribute to this specificity of CREB such as chromatin structure, methylation status at CRE sites, presence of accessory proteins, and transcription factor cooperativity. Evidently, further studies are needed to clarify brain regional differences in the regulatory mechanisms governing BER.

Interestingly, no CREB‐binding to the predicted CRE site in the promoter of the *Neil2* gene was observed in our CREB‐binding study. This is despite a strong tendency toward NEIL2 upregulation upon BDNF treatment in both mouse and rat primary hippocampal neurons and a significant positive correlation between *NEIL2* and *BDNF* expression in the human HC. This suggests that NEIL2 could be regulated by BDNF independent of CREB binding to its promoter, consistent with BDNF signaling causing activation of a number of different transcription factors besides CREB such as NFκB (Marini et al., 2008). Kinslow et al. has partially characterized the promoter region of the *NEIL2* gene and in addition to CREB, the *NEIL2* promoter also contains predicted binding sites for AP‐1, Sp‐1, NFκB, YY1, and PEA3 providing additional potential regulatory mechanisms for NEIL2 expression (Kinslow et al., 2010).

We studied the methylation status at a subset of the CRE sites in the aged mouse brain, since methylation strongly affects the affinity of CREB for the CRE site (Kitsera et al., 2017; Zhang et al., 2005). We did not detect methylation at the CRE site in the promoter of either *Ape1* or *Polb*, suggesting that methylation is not involved in CREB‐dependent regulation of these genes. On the other hand, we observed a medium to high methylation frequency for the CRE site in the promoter of *Neil1*, *Ung*, and *Ogg1*, respectively. It is possible that these sites are regulated by methylation during aging. Indeed, downregulation in gene expression with age has been associated with promoter methylation for *Ogg1* and *Neil1* in the aging mouse brain (Langie et al., 2017). In this way, our findings are in line with the previous findings reporting increased methylation of the *Ogg1* promoter in DNA isolated from mouse brains with age, where the highest peak of methylation was in 28 months old mice as compared to 3 months old mice. In contrast, the averaged methylation status of the *Neil1* promoter was not significantly affected by age, although the methylation at a few CpG sites was changed with age. In many cases, different brain regions have distinct patterns of methylation and regional as well as cell‐type‐specific differences in the methylation status of the CRE sites investigated here cannot be ruled out, since our analysis was conducted on whole brains. However, it is also possible that the highly methylated CRE sites observed here, although able to bind CREB in an unmethylated state in vitro, are not functional in vivo. Accordingly, it has previously been shown that in vitro methylation of the *UNG* promoter strongly reduces promoter activity (Haug et al., 1996). However, only a narrow region of the 5′ CpG island constituting the potential transcription factor binding site seems to be invariable methylation‐free in vivo, suggesting that methylation might not be involved in regulating *UNG* expression. Interestingly, our analysis show that UNG expression is increased with age and negatively correlates with BDNF expression in the human brain (EC, PCG, SFG). This finding again indicates an alternative regulatory axis of *UNG/Ung* compared with the other BER genes largely showing a positive correlation with BDNF expression. Additional studies are needed in order to understand which of the CRE sites identified in our analysis are functional in vivo and whether tissue‐specific differences in CREB occupancy at functional sites exist in the brain.

Finally, based on the identified link between BDNF and BER, we investigated the outcome of reduced BDNF on the DNA repair capacity in 4 months old *Bdnf* ^+/−^ mice. *Bdnf* heterozygosity in mice is known to result in memory and learning deficits and increased susceptibility to stress‐induced oxidative damage (Geist et al., 2017; Hacioglu et al., 2016; Linnarsson et al., 1997). Notably, *Bdnf* heterozygosity was associated with reduced APE1 and POLB protein levels in the HC, in agreement with our observation in BDNF stimulated hippocampal neurons. Surprisingly, protein levels of APE1 and POLB were not altered in cortex, thus supporting the idea that regulation of BER by BDNF in the brain is region‐specific. We did not detect significant changes in the DNA repair activity or the level of DNA damage in HC. Thus, we speculate that compensatory mechanisms might be in play including parallel regulatory mechanisms, transcription factor competition for CRE sites (Steven et al., 2020), other DNA repair enzymes serving as backups (Krokan & Bjoras, 2013) or upregulation of repair activity by post‐translational modifications (Carter & Parsons, 2016). Moreover, *Bdnf* ^+/−^ mice are known to display age‐dependent deficits in learning and behavior as well as changes in gene expression (Endres & Lessmann, 2012; Petzold et al., 2015; Saylor et al., 2006) suggesting that mechanisms exist that can compensate for BDNF deficiency in young but not old animals.

**FIGURE 6:**
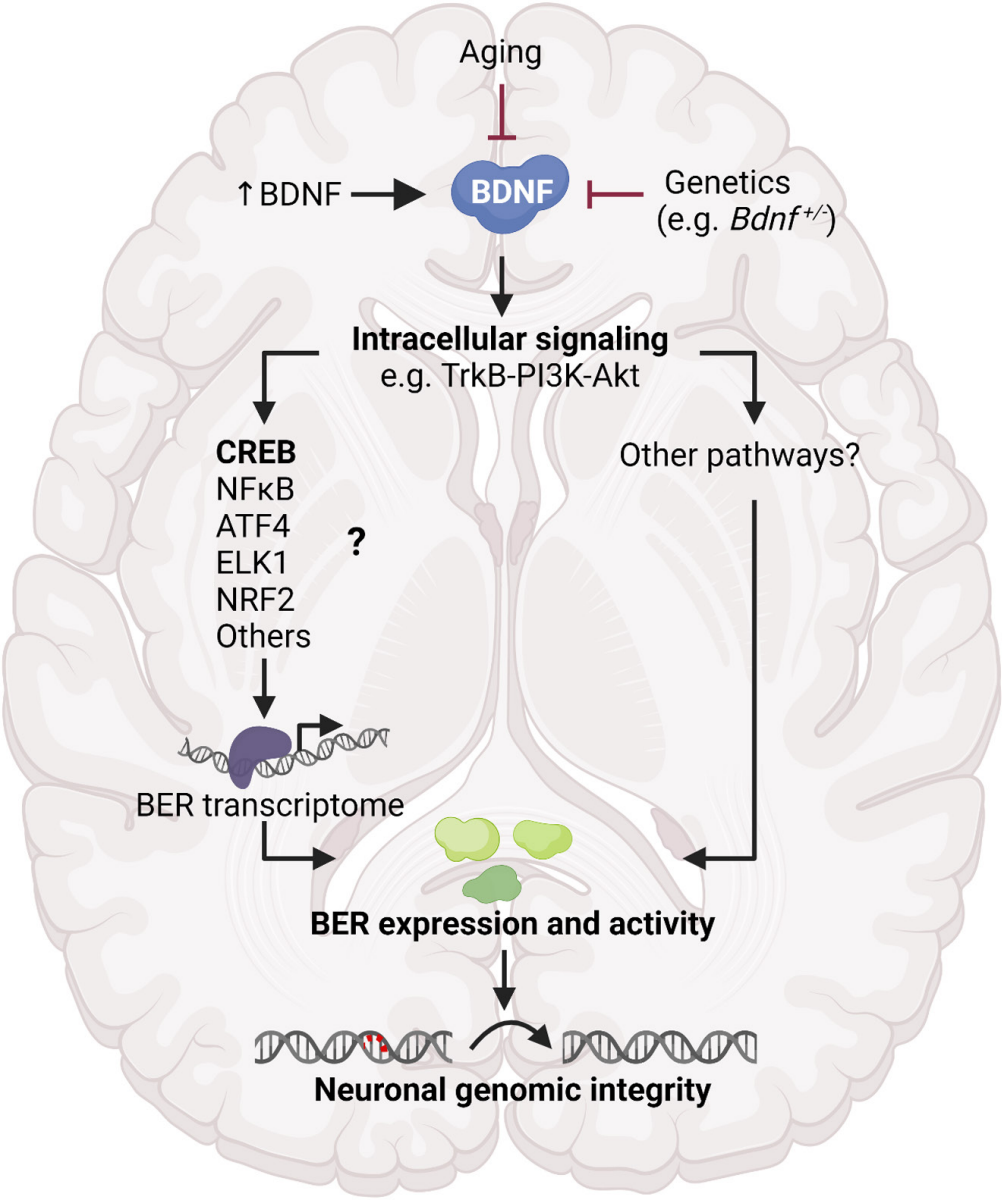
Proposed model of the regulatory BDNF‐BER axis in the brain Mature BDNF binds to its cellular receptor TrkB initiating the activation of intracellular signaling cascades such as the PI3K‐Akt pathway. This in turn leads to the phosphorylation, and hereby activation, of the transcription factor CREB as well as activation of other transcription factors including NFκB, ATF4, ELK1, and NRF2. Activated transcription factors bind to their recognition sites (e.g., CRE sites for CREB) in the promoter of core BER genes and stimulate their transcription. Thereby, CREB and/or other BDNF‐activated transcription factors, positively regulates the BER transcriptome, resulting in increased BER protein levels and activity, hereby contributing to DNA repair in the neurons. However, there might also be alternative pathways by which BDNF can regulate BER independent of transcription of BER genes. In this study we show that by treating primary hippocampal neurons with BDNF (↑BDNF) pCREB and pAKT increases, and so do BER expression and activity. On the contrary, when having less BDNF, as seen in *Bdnf* ^*+/−*^ mice, BER expression is reduced in a brain‐region‐specific manner. Furthermore, we demonstrate that in the human brain, BDNF in parallel with the BER transcriptome decreases with age, suggesting that BDNF contributes to the age‐associated genomic instability. Created with Biorender.com.

In summary, our findings in combination with previous studies propose that BDNF participates in regulation of transcription of core BER genes, and hereby contribute to genomic repair and stability in the brain (Figure 6, proposed model). BDNF might regulate BER via CREB at a transcriptional level as our in vitro analysis of CREB binding to BER promoters suggests. However, other BDNF‐regulated transcription factors including NFκB, activating‐transcription factor 4 (ATF4), ETS Like‐1 protein (ELK1), and nuclear factor erythroid 2‐related factor 2 (NRF2) might also be involved (Caviedes et al., 2017; Ishii et al., 2019; Kajiya et al., 2008; Liu, Amar, et al., 2018) as well as regulation via other pathways independent of transcriptional control of BER genes.

Reduced levels of BDNF, as observed in the *Bdnf* ^+/−^ mice and during human brain aging, of course have other effects in addition to the direct connection to BER based on its involvement in regulating various processes. However, this does not mean that the BDNF‐BER regulatory axis is not important. Accordingly, decreased or complete loss of functional BER, especially in the neurons, affects both cognitive performance, health span, and lifespan (Hou et al., 2018; Misiak et al., 2017). The BDNF‐BER regulatory axis likely contributes to neuronal function, survival, and protection against oxidative stress. Accordingly, we propose that one of the important roles of BDNF in neurons is due to its function as a transcriptional regulator of BER gene expression and hereby DNA repair. Further studies are needed in order to clarify the physiological effects of this BDNF‐mediated regulation of BER in the aging brain.

## AUTHORS' CONTRIBUTION

S.L., C.M.H., and T.S. conceived and contributed to the design of the study. M.R., C.B.V., L.S.L., G.A.P., N.B., and C.W.C. provided study material and/or assembly of data. S.L., C.M.H., A.Y., S.A., and V.T. performed the experiments. S.L., C.M.H., K.L., and N.B. analyzed and interpreted the data. Manuscript was written by S.L., C.M.H., and T.S. with the help of the other co‐authors. All authors reviewed and approved the manuscript.

## CONFLICT OF INTEREST STATEMENT

The authors declare no conflicts of interest.

## Supporting information


Data S1

Figures S1–S8

Tables S1–S9

## Data Availability

The data that support the findings of this study are available from the corresponding author upon reasonable request. Microarray data is available in the Gene Expression Omnibus database (www.ncbi.nlm.nih.gov/geo) with accession number GSE11882.
