# Supplementary material for: The role of aging and brain‐derived neurotrophic factor signaling in expression of base excision repair genes in the human brain
Source: Aging Cell. 2023 Jun 19;22(9):e13905. doi: 10.1111/acel.13905 (PMC10497833; doi:10.1111/acel.13905)
Supplement: Supplementary file 1 — Data S1 [file ACEL-22-e13905-s003.pdf]

Supplementary materials and methods

DNA purification and bisulfite sequencing

Whole mouse brain tissue was homogenized using a TissueLyser with stainless steel beads (Qiagen) for 3x30s. Three hundred microliter lysis buffer (50 mM Tris pH 8, 100 mM EDTA, 100 mM NaCl, 1% SDS) and 30 µL 10 mg/mL proteinase K (Qiagen) was added to the homogenized tissue and incubated at 55°C for 12-18 hr. Subsequently, 180 µL 6M NaCl was added followed by vigorous vortexing for 15-20s and centrifugation for 15 min at 20,000xg at 4°C. The DNA was pelleted by addition of 500 µL isopropanol to the supernatant followed by centrifugation at 20,000xg for 10 min at 4°C. The DNA pellet was washed in 70% ethanol, dried briefly, and resuspended in 100 µL 1xTE buffer and 1 µL 10 mg/mL RNase A. DNA concentrations were measured on a NanoDrop Lite Spectrophotometer (Thermo Scientific).

Bisulfite sequencing was used to evaluate the level of methylation at the CpG position in selected CRE sites in the promoter of BER genes. Selected CRE sites and primers used for PCR and sequencing are listed in Table S9. Bisulfite conversion of the purified DNA was performed using the EZ DNA Methylation Gold™ Kit (Zymo Research/Nordic Bioscience) according to manufacturer's protocol. The subsequent PCR was performed with the PyroMark PCR Kit (Qiagen). PCR reactions contained 50 ng bisulfite treated DNA as template, 0.2 µM forward primer, 0.2 µM reverse primer, 1 mM $MgCl_2$, 1x CoralLoad Concentrate, and 1x PyroMark PCR Master Mix. The PCR cycling conditions were as follows: 1 cycle of 95°C for 15 min, 45 cycles of 94°C for 30s, 56°C for 30s, and 72°C for 30s, and 1 cycle of 72°C for 10 min. PCR products were run on 2% agarose gels, purified using the MinElute® Gel Extraction Kit (Qiagen) according to manufacturer's protocol, and sequenced (GATC, Eurofins Genomics) using the reverse primers listed in Table S9. The estimated percentage methylation at each CpG position was calculated as described in (Jiang *et al.* 2010).

Primary rat neuronal cultures and Western blot assay

Primary cultures of dissociated hippocampal neurons were prepared from E18 WT Sprague-Dawley rats as described previously (Pike *et al.* 1993). Cells were maintained in complete medium, defined as serum-free DMEM supplemented with B27, Glutamax and penicillin/streptomycin (all culture reagents from Invitrogen). Cell cultures were treated with 50 ng/ml BDNF as specified.

Western blot assay was performed essentially as described in (Smith *et al.* 2014). Briefly, 20 µg WCE was boiled in SDS NuPage Loading Dye (Novus Life Technologies) before loading on a 10% TA polyacrylamide gel. The gel was run for 1hr 40 min at 150 V in 1x TA running buffer. The gel was rinsed in ddH_2O before transfer at 100 V for 30min. After transfer, the membrane was rinsed in TBS-T,

blocked with 5% low fat skim milk-TBS-T, followed by incubation with primary antibody ON at 4°C. The primary antibodies used were rabbit anti-NEIL2 (Abcam #ab180576) 1:5000, rabbit anti-APE1 (Thermo Scientific #PA5-29157) 1:2000, rabbit anti-phospho-AKT (Ser473) (Cell signaling #CST9271S) 1:1000, mouse anti-Actin (Sigma #A2228) 1:20,000, and rabbit anti-FEN1 Abcam #ab153825) 1:500. After incubation with primary antibody the membrane was washed in TBS-T, then incubated with secondary antibody for 1 hr at RT, washed in TBS-T and detected with ECL prime (Amersham). Secondary antibodies used were anti-rabbit IgG horseradish peroxidase-linked (GE Healthcare #NA934) 1:5000 and anti-mouse IgG horseradish peroxidase-linked (GE Healthcare #NA931) 1:5000. Because of very similar size of proteins, membranes were, when necessary, stripped with Restore PLUS western blot stripping buffer (Thermo Scientific) before re-probing with antibodies against other antigens. Western blots were quantified by use of ImageJ software.

Supplementary references

Jiang, M., Y. Zhang, J. Fei, X. Chang, W. Fan *et al.*, 2010 Rapid quantification of DNA methylation by measuring relative peak heights in direct bisulfite-PCR sequencing traces. Lab Invest 90**:** 282-290.

Pike, C. J., D. Burdick, A. J. Walencewicz, C. G. Glabe and C. W. Cotman, 1993 Neurodegeneration induced by beta-amyloid peptides in vitro: the role of peptide assembly state. J Neurosci 13**:** 1676-1687.

Smith, E. D., G. A. Prieto, L. Tong, I. Sears-Kraxberger, J. D. Rice *et al.*, 2014 Rapamycin and interleukin-1β impair brain-derived neurotrophic factor-dependent neuron survival by modulating autophagy. J Biol Chem 289**:** 20615-20629.
