# Supplementary material for: The role of aging and brain‐derived neurotrophic factor signaling in expression of base excision repair genes in the human brain
Source: Aging Cell. 2023 Jun 19;22(9):e13905. doi: 10.1111/acel.13905 (PMC10497833; doi:10.1111/acel.13905)
Supplement: Supplementary file 2 — Figures S1–S8 [file ACEL-22-e13905-s002.pdf]

EC

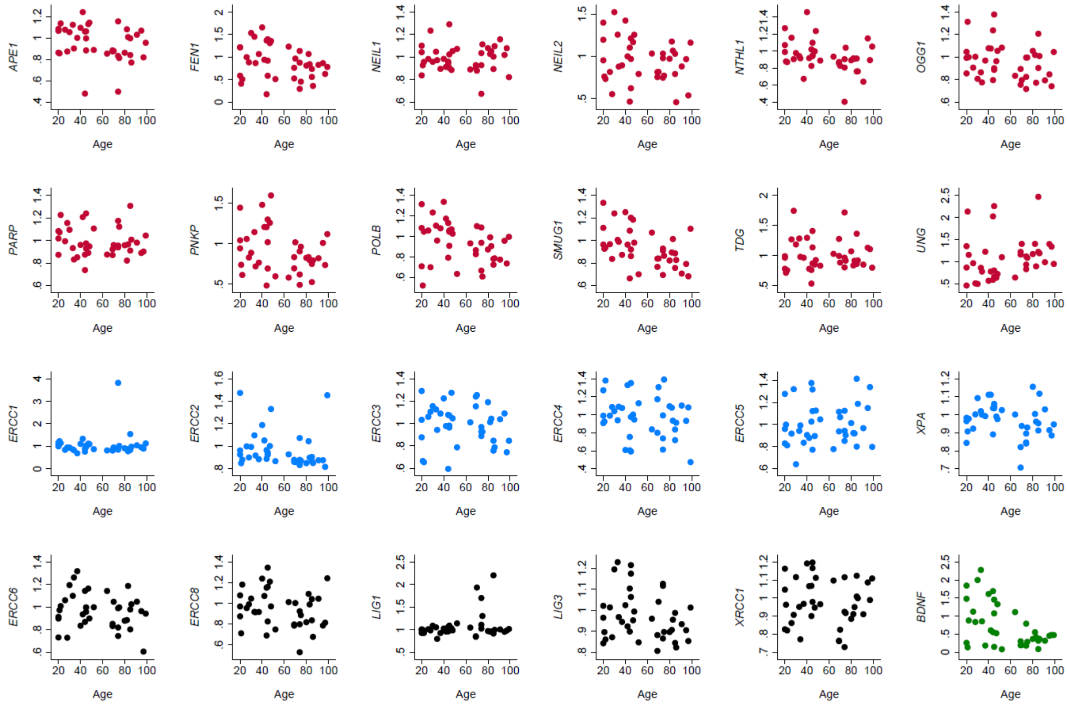
APE1
FEN1
NEIL1
NEIL2
NTHL1
OGG1
PARP
PNKP
POLB
SMUG1
TDG
UNG
ERCC1
ERCC2
ERCC3
ERCC4
ERCC5
XPA
ERCC6
ERCC8
LIG1
LIG3
XRCC1
BDNF
Age

HC

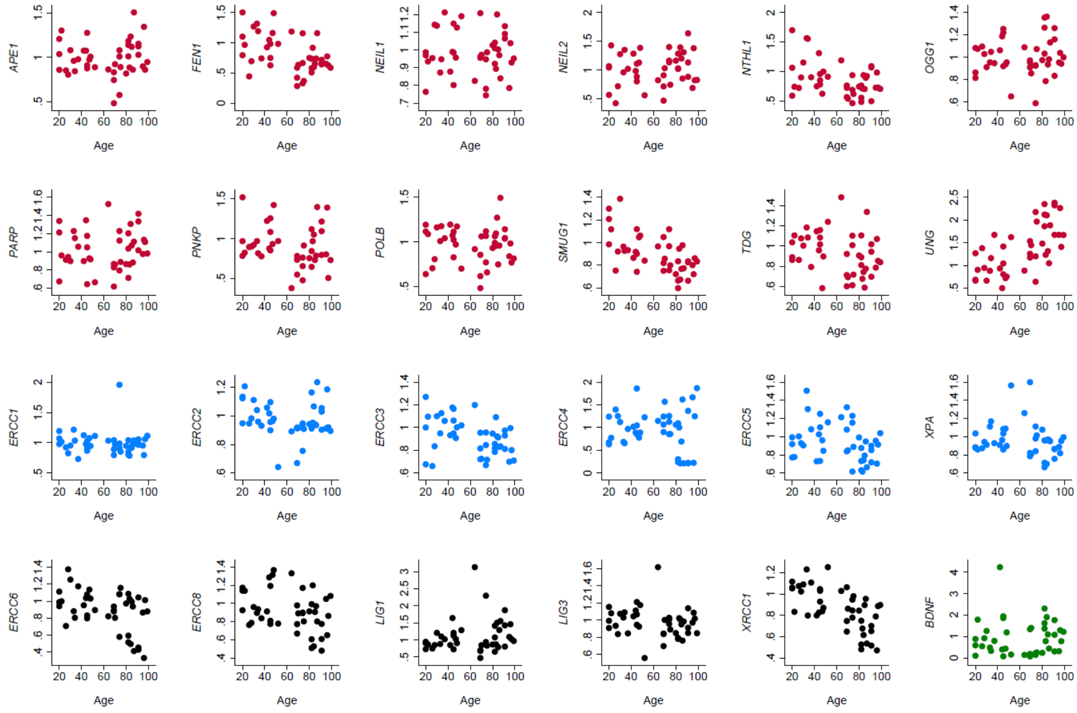
APE1
FEN1
NEIL1
NEIL2
NTHL1
OGG1
PARP
PNKP
POLB
SMUG1
TDG
UNG
ERCC1
ERCC2
ERCC3
ERCC4
ERCC5
XPA
ERCC6
ERCC8
LIG1
LIG3
XRCC1
BDNF
Age

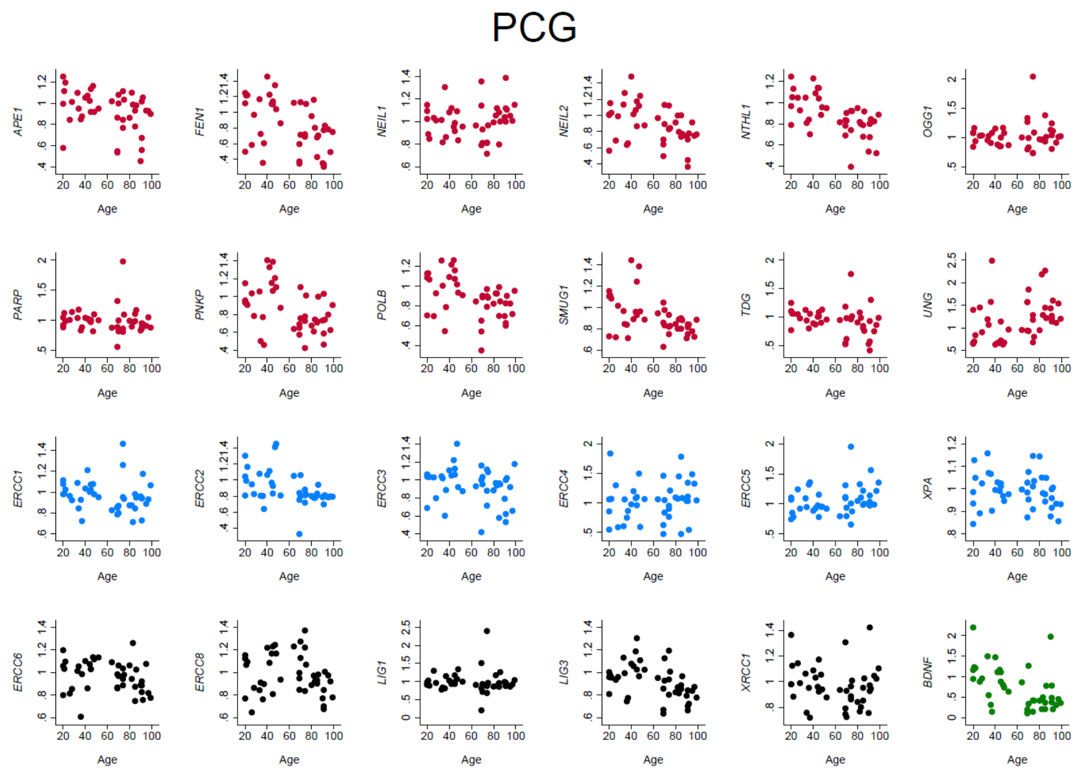

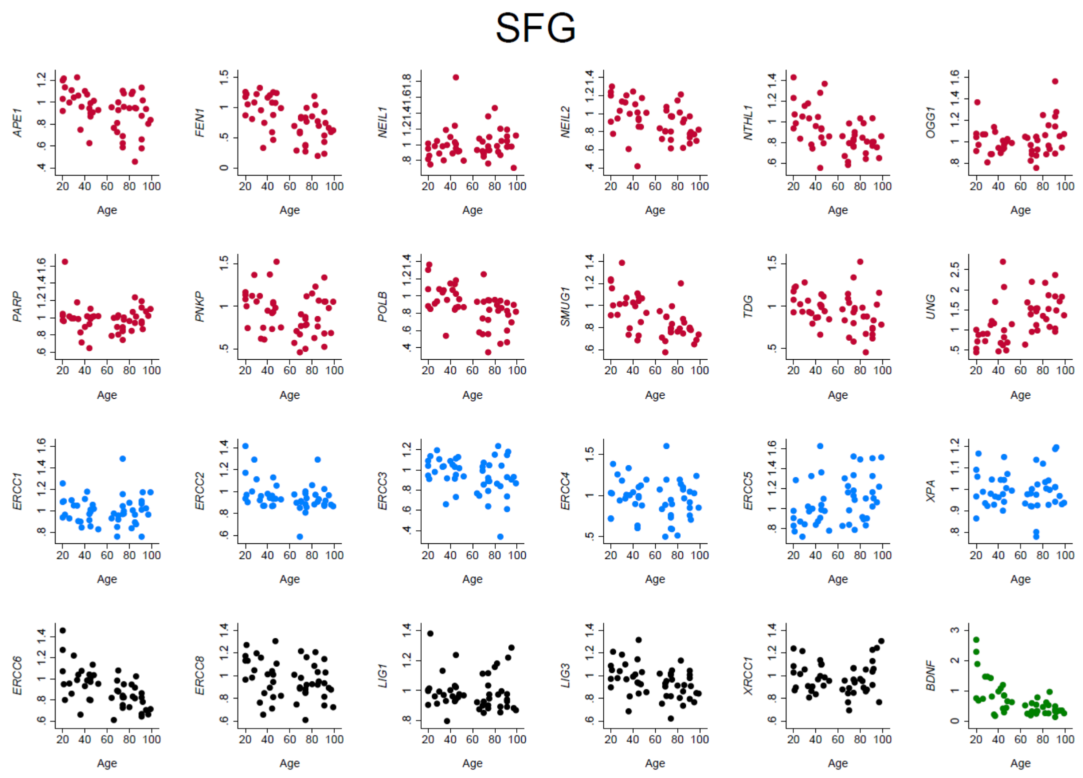

Figure S1 Expression of NER and BER genes plotted against age A) EC: entorhinal cortex (N=39); B) HC: hippocampus (N=40); C) PCG: postcentral gyrus (N=43); D) SFG: superior frontal gyrus (N=48). Individuals aged 20-99 years. Red: BER genes. Blue: NER genes. Black: Genes shared between BER and NER. Green: BDNF. Values on Y-axis correspond to relative expression.

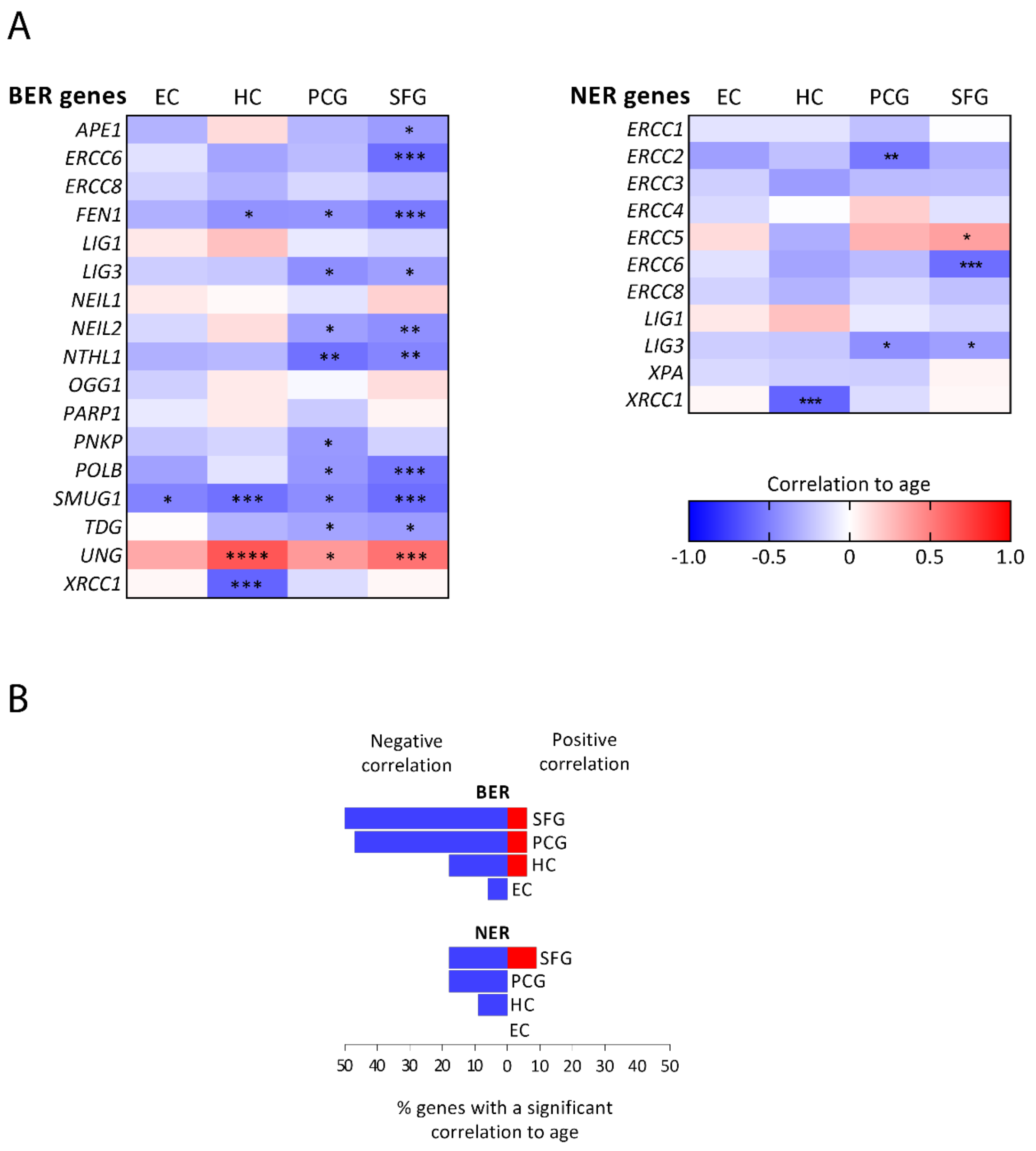

Figure S2 Correlation between age and expression of DNA repair genes involved in BER and NER in four human brain regions EC: entorhinal cortex (N=39); HC: hippocampus (N=40); PCG: postcentral gyrus (N=43); SFG: superior frontal gyrus (N=48). Individuals aged 20-99 years. A) Spearman's rank correlation coefficient between BER and NER genes and age as a continuous variable. Benjamini-Hochberg correction for multiple testing. *: $p \leq 0.05$; **: $p \leq 0.01$; *** $p \leq 0.001$; ****: $p \leq 0.0001$. B) Percentage of BER and NER genes with a significant positive or negative correlation (as computed in A) to age in the four brain regions. Genes involved in both pathways are included in both the BER and NER analysis. Changes were considered significant at $p<0.05$.

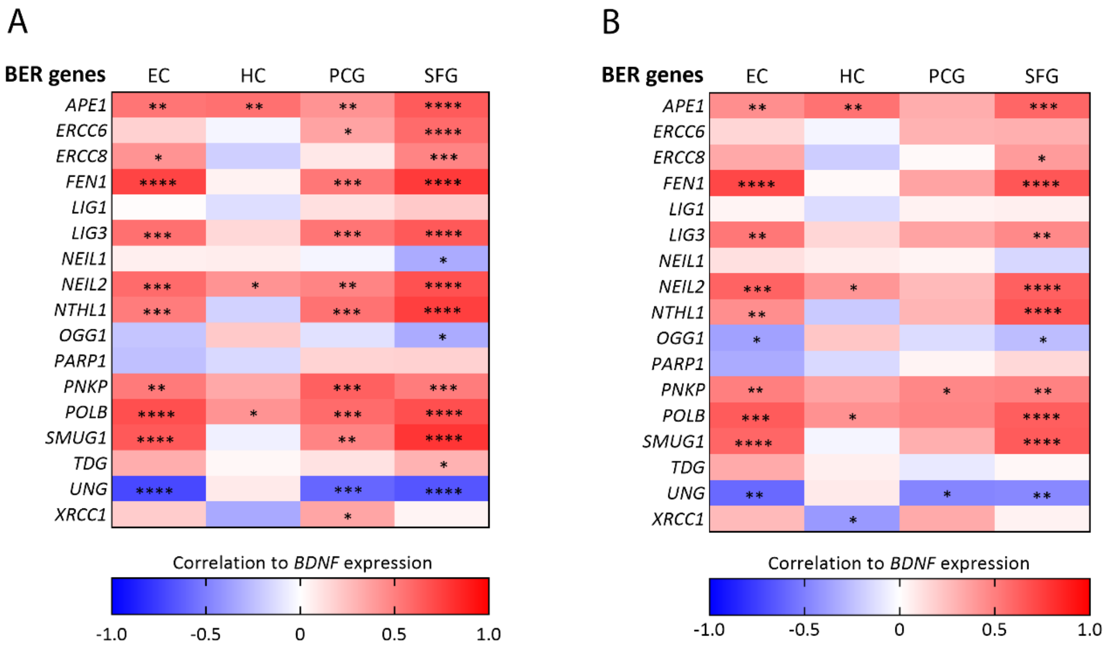

Figure S3 Correlation between expression of *BDNF* and BER genes in four human brain regions with and without age effects adjustment EC: entorhinal cortex (N=38); HC: hippocampus (N=41); PCG: postcentral gyrus (N=42); SFG: superior frontal gyrus (N=47). Individuals aged 20-99 years. A) Spearman's rank correlation coefficient. No adjustment for age effects. B) Partial Spearman's rank correlation coefficient adjusted for age. Benjamini-Hochberg correction for multiple testing. *: $p \leq 0.05$; **: $p \leq 0.01$; *** $p \leq 0.001$; ****: $p \leq 0.0001$.

EC

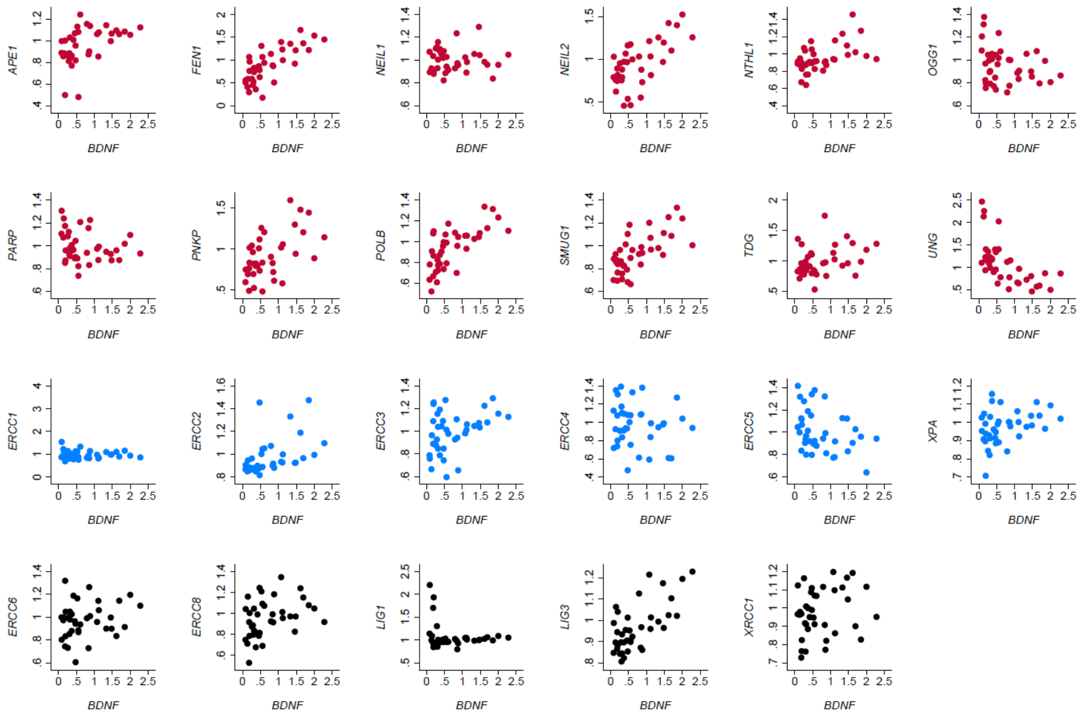
APE1
FEN1
NEIL1
NEIL2
NTHL1
OGG1
PARP
PNKP
POLB
SMUG1
TDG
UNG
ERCC1
ERCC2
ERCC3
ERCC4
ERCC5
XPA
ERCC6
ERCC8
LIG1
LIG3
XRCC1
BDNF

HC

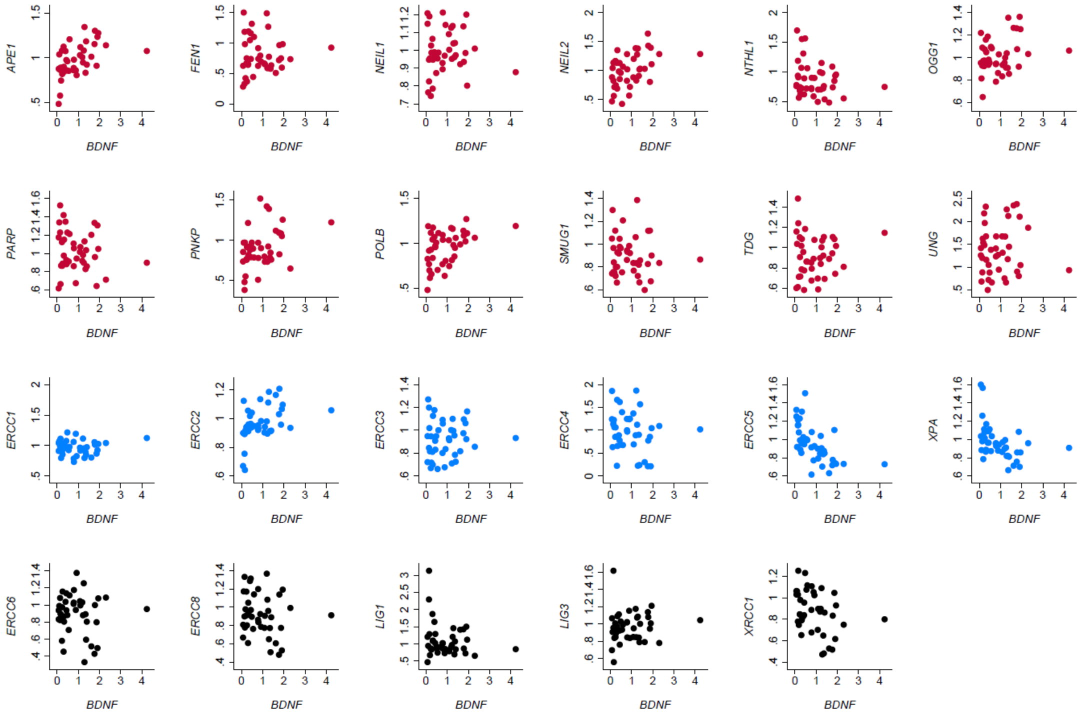
APE1
FEN1
NEIL1
NEIL2
NTHL1
OGG1
PARP
PNKP
POLB
SMUG1
TDG
UNG
ERCC1
ERCC2
ERCC3
ERCC4
ERCC5
XPA
ERCC6
ERCC8
LIG1
LIG3
XRCC1
BDNF

PCG

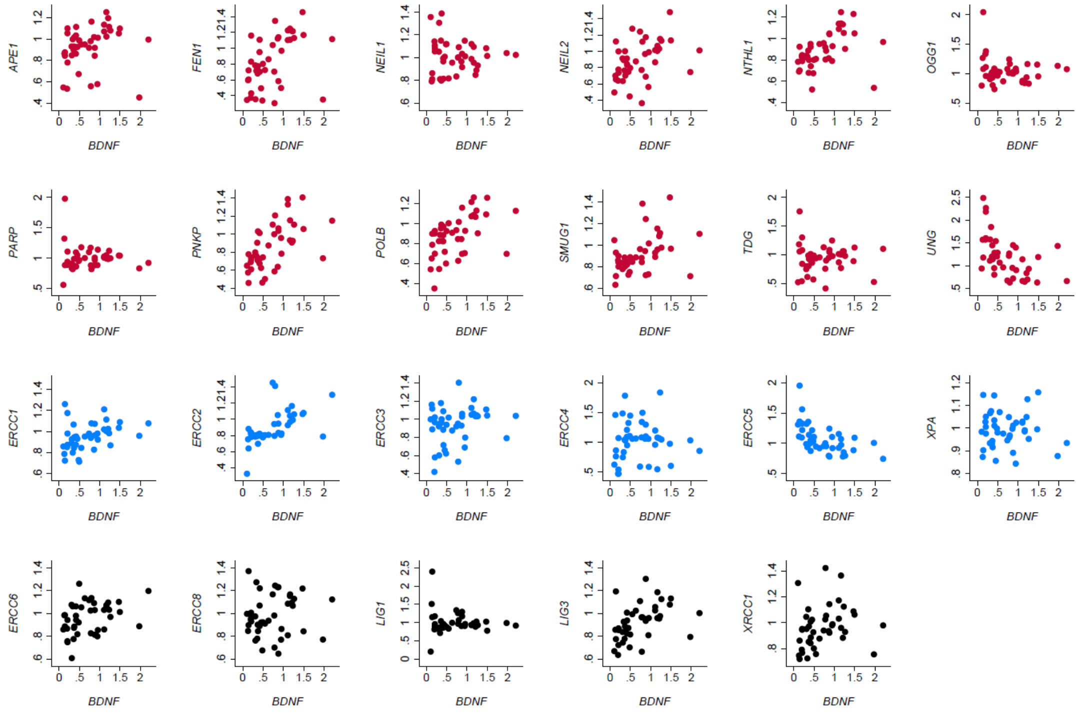

SFG

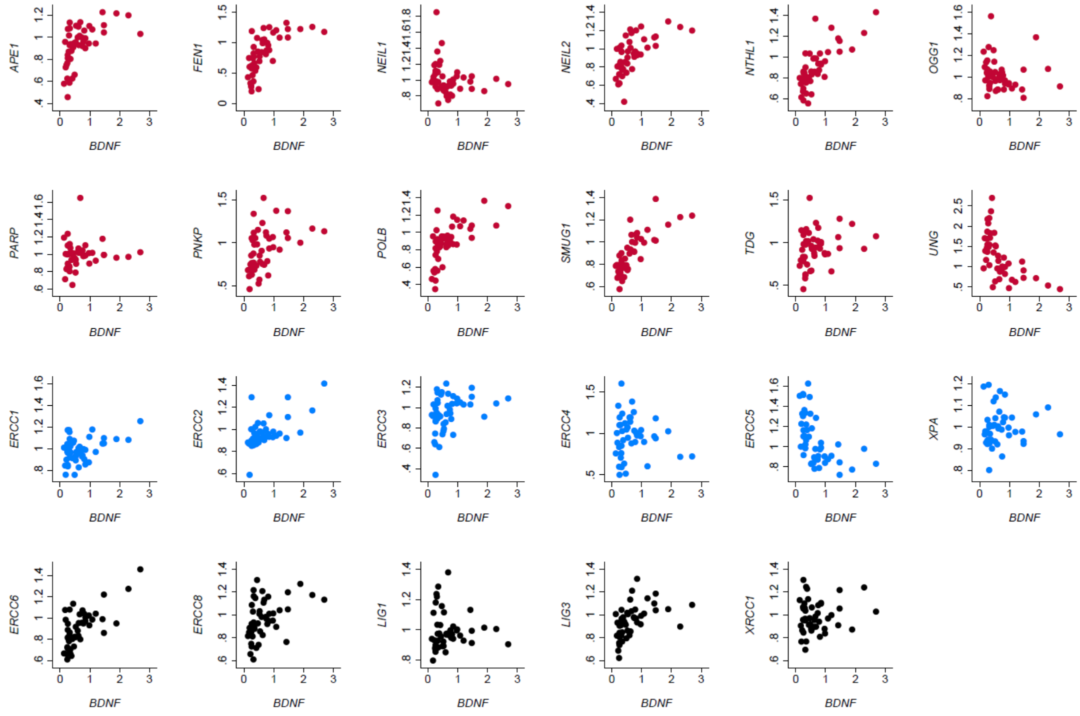

Figure S4 Expression of NER and BER genes plotted against *BDNF* expression A) EC: entorhinal cortex (N=38) B) HC: hippocampus (N=41) C) PCG: postcentral gyrus (N=42) D) SFG: superior frontal gyrus (N=47). Individuals aged 20-99 years. Red: BER genes. Blue: NER genes. Black: Genes shared between BER and NER. Values on Y- and X-axis correspond to relative expression.

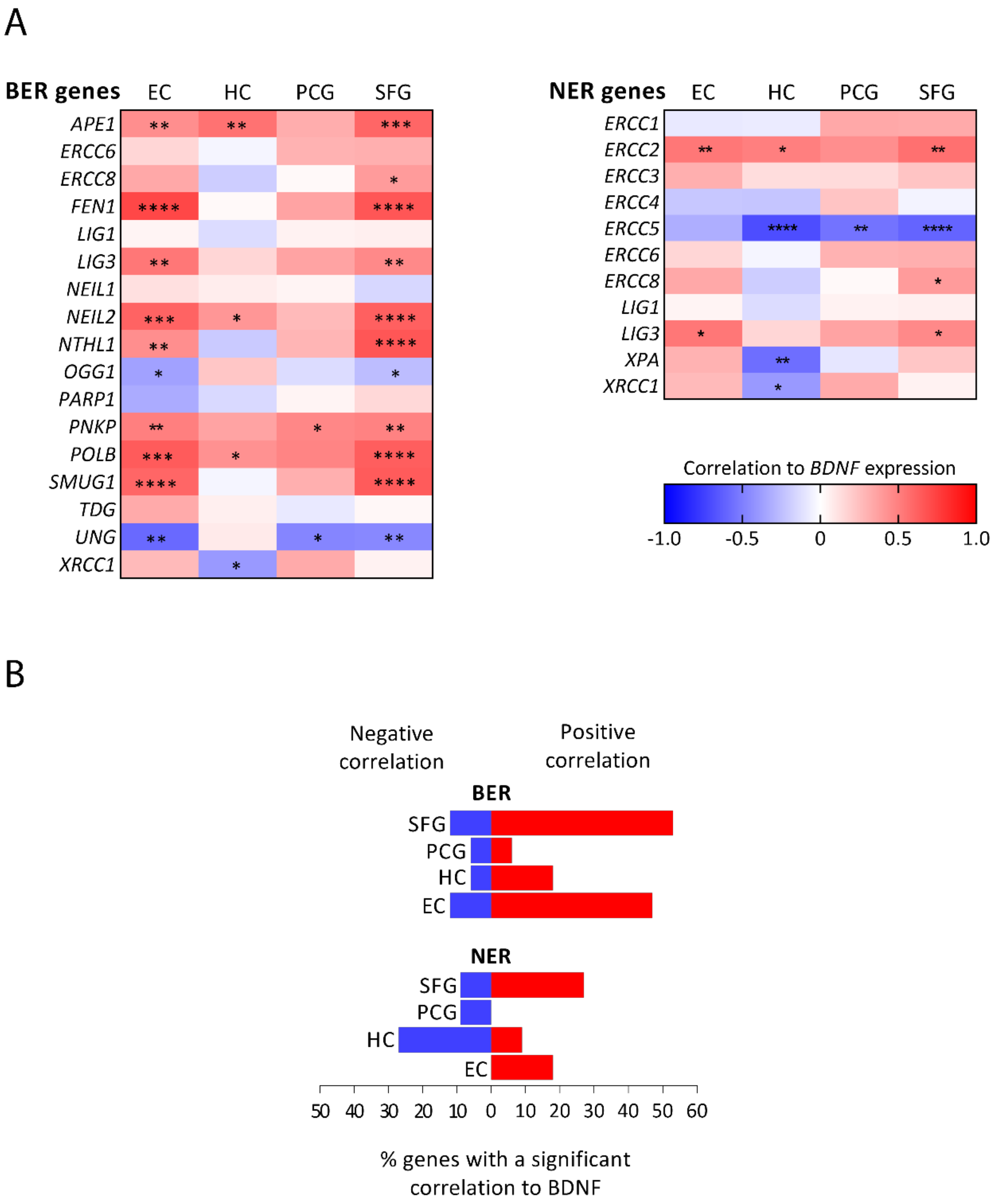

Figure S5 Correlation between expression of *BDNF* and DNA repair genes involved in BER and NER in four human brain regions EC: entorhinal cortex (N=38); HC: hippocampus (N=41); PCG: postcentral gyrus (N=42); SFG: superior frontal gyrus (N=47). Individuals aged 20-99 years. A) Partial Spearman's rank correlation coefficient adjusted for age for BER and NER genes, respectively. Benjamini-Hochberg correction for multiple testing. *: p≤0.05; **: p≤0.01; *** p≤0.001; ****: p≤0.0001. B) Percentage of BER and NER genes with a significant positive or negative correlation (as computed in A) to *BDNF* expression in the four brain regions. Genes involved in both pathways are included in both the BER and NER analysis. Changes were considered significant at p<0.05.

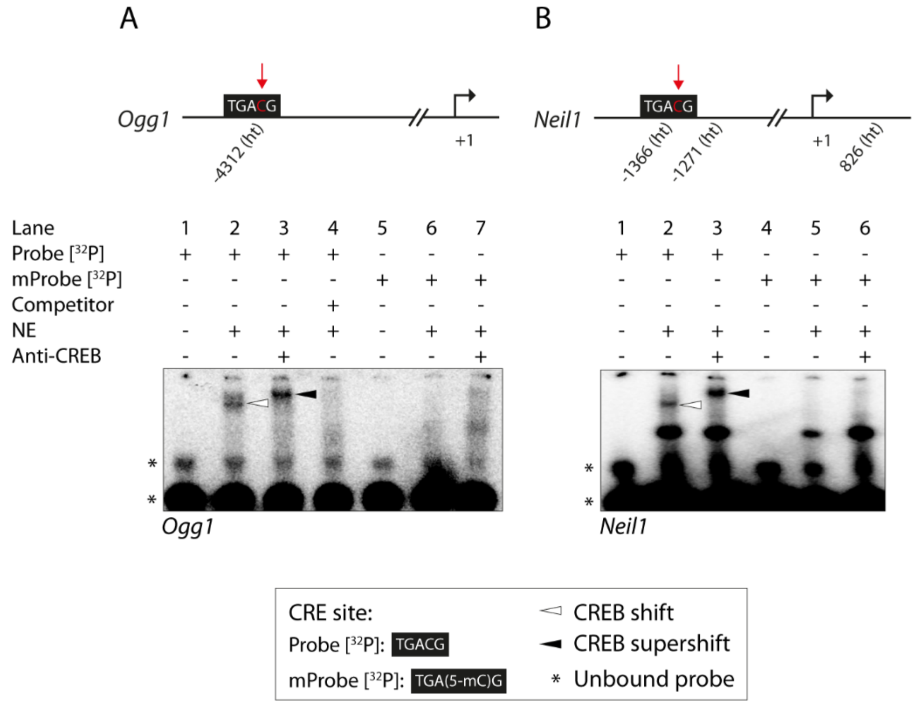

Figure S6 Methylation at the CpG position in CRE site prevents CREB binding at two BER promoters Upper panel: Position of predicted CRE sites in the promoter of the two selected BER genes, *Ogg1* and *Neil1*. These genes were selected as they were shown to exhibit methylation at the CpG position in the predicted CRE site in the mouse brain (see Fig. S7). CRE site in black box: CRE site investigated in EMSA. Red arrow/letter marks the C in the CRE site either methylated or unmethylated in the analysis. Lower panel: EMSA with mouse brain nuclear extract (NE). Assays conducted with ^{32}P-labelled probes corresponding to potential CRE sites based on *in silico* predictions and immediate up- and downstream flanking sequence of the BER promoters. A) EMSA with *Ogg1* probe. Lane 1: ^{32}P-labelled probe without NE. Lane 2: ^{32}P-labelled probe + NE. White arrowhead indicates CREB shift. Lane 3: ^{32}P-labelled probe + NE + anti-CREB antibody. Black arrowhead indicates CREB super shift. Lane 4: ^{32}P-labelled probe + NE + competitor (unlabelled POLB probe in molar excess). Lane 5-7: identical to lane 1-3 except a ^{32}P-labelled methylated probe (mProbe) with 5-methylcytosine at CpG position in CRE site on both strands was used as probe. * marks unbound probe. B) EMSA with *Neil1* probe. Lane 1: ^{32}P-labelled probe without NE. Lane 2: ^{32}P-labelled probe + NE. White arrowhead indicates CREB shift. Lane 3: ^{32}P-labelled probe + NE + anti-CREB antibody. Black arrowhead indicates CREB super shift. Lane 4-6: identical to lane 1-3 except a ^{32}P-labelled methylated probe (mProbe) with 5-methylcytosine at CpG position in CRE site on both strands. * marks unbound probe. The sequence of the probes can be found in Table S3.

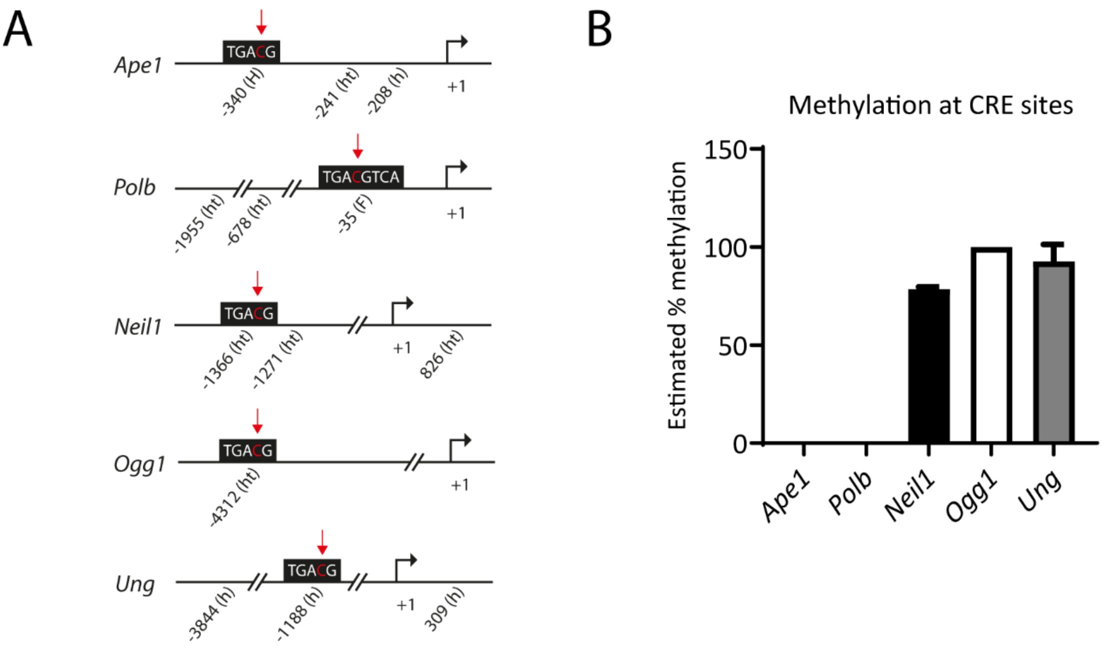

Figure S7 Methylation status at CpG position in CRE site of BER promoters in the aged mouse brain The level of methylation was estimated at selected CRE sites in promoter region of chosen BER genes in the mouse genome. A) Position of selected CRE sites relative to TSS in the methylation analysis. CRE sites investigated are marked in a black box and C in the CpG position of the CRE side are marked in red. B) DNA was isolated from the brain of middle-aged to old mice (11-28 months, n=9) and methylation status estimated by bisulfite sequencing at the CpG position in the selected CRE sites. Values are mean and SEM.

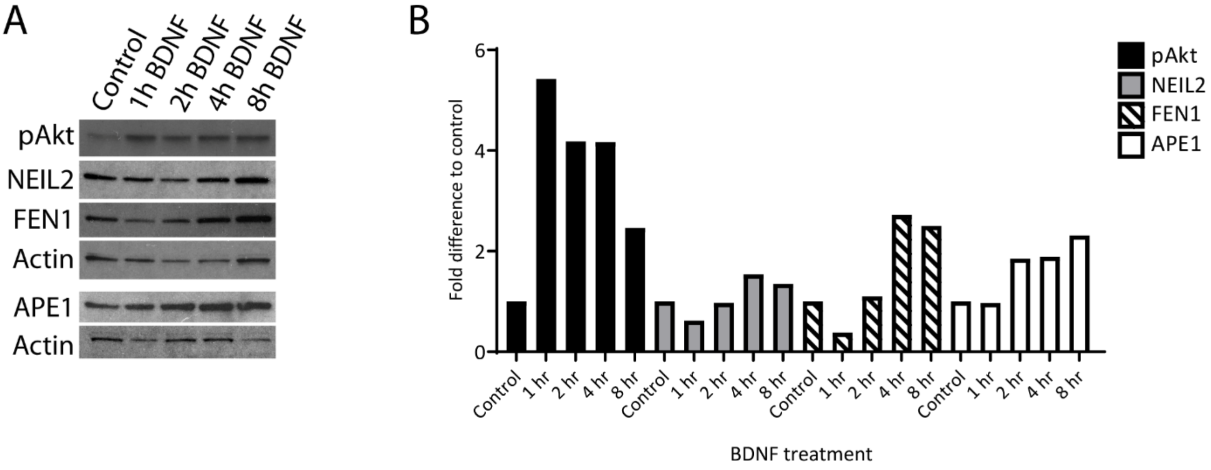

Figure S8 Activation of intracellular signaling and increased BER protein expression after BDNF treatment of rat primary hippocampal neurons Primary neurons were treated with 50 ng/mL BDNF for indicated time periods or control without BDNF treatment and activation of intracellular signalling and BER protein expression evaluated by immunoblotting, n=1 independent culture. Values are relative to Actin level. All values are fold difference compared to control.
