# Supplementary material for: The role of aging and brain‐derived neurotrophic factor signaling in expression of base excision repair genes in the human brain
Source: Aging Cell. 2023 Jun 19;22(9):e13905. doi: 10.1111/acel.13905 (PMC10497833; doi:10.1111/acel.13905)
Supplement: Supplementary file 3 — Tables S1–S9 [file ACEL-22-e13905-s001.pdf]

	**Young**	**Aged**
**Age range**	20-59	69-99
**Population number, N**	22	33
**Age, mean (SD)**	35.4 (10.3)	83.2 (9.0)
**Sex frequency (%)**		
**Female**	45.5	54.5
**Male**	54.5	45.5
**N per brain region**		
**EC**	21	17
**HC**	18	24
**PCG**	19	23
**SFG**	22	24

Table S1 Characteristics for individuals in the microarray analysis EC: entorhinal cortex; HC: hippocampus; PCG: postcentral gyrus; SFG: superior frontal gyrus.

	Brain region	EC	HC	PCG	SFG
**Probeset(s)**	**BER genes**				
**2010027_s_at**	***APE1***	0.90	0.99	0.87*	0.87*
**230108_at**	***ERCC6***	0.92	0.81*	0.93	0.83***
**1554882_at, 1554883_a_at, 205162_at**	***ERCC8***	0.89	0.83*	0.96	0.94
**204767_s_at, 204768_s_at**	***FEN1***	0.74*	0.66****	0.68***	0.66****
**202726_at**	***LIG1***	1.16	1.09	0.98	0.99
**204123_at, 207348_s_at, 1555169_at**	***LIG3***	0.93	0.92	0.85**	0.90*
**219396_s_at, 241477_at**	***NEIL1***	0.98	0.98	1.01	1.02
**226585_at**	***NEIL2***	0.88	1.06	0.79**	0.87*
**209731_at**	***NTHL1***	0.86*	0.74**	0.78****	0.81***
**205760_s_at, 205301_s_at**	***OGG1***	0.91	1.01	1.06	1.03
**208644_at**	***PARP1***	0.98	1.01	0.99	0.97
**218961_s_at**	***PNKP***	0.81*	0.85	0.74***	0.87
**203616_at**	***POLB***	0.86*	0.95	0.79***	0.78***
**234708_at, 223684_s_at**	***SMUG1***	0.84**	0.82***	0.83**	0.80****
**203743_s_at**	***TDG***	1.02	0.85**	0.87	0.89
**202330_s_at**	***UNG***	1.23	1.67****	1.33*	1.50***
**203655_at**	***XRCC1***	0.94	0.75****	0.94	0.97
	**NER genes**				
**203720_s_at, 203719_at, 228131_at**	***ERCC1***	1.13	0.99	0.94	1.00
**213468_at**	***ERCC2***	0.92	0.96	0.79***	0.92*
**202176_at**	***ERCC3***	0.98	0.85***	0.89	0.91
**210158_at**	***ERCC4***	0.95	0.92	1.05	0.92
**202414_at**	***ERCC5***	1.03	0.88	1.12	1.14*
**230108_at**	***ERCC6***	0.92	0.81*	0.93	0.83***
**1554882_at, 1554883_a_at, 205162_at**	***ERCC8***	0.89	0.83*	0.96	0.94
**202726_at**	***LIG1***	1.16	1.09	0.98	0.99
**204123_at, 207348_s_at, 1555169_at**	***LIG3***	0.93	0.92	0.85**	0.90*
**205672_at, 232967_at, 1559515_at**	***XPA***	0.93*	0.92	0.98	0.99
**203655_at**	***XRCC1***	0.94	0.75****	0.94	0.97
	***BDNF* gene**				
**239367_at, 206382_at**	***BDNF***	0.36***	0.92	0.48***	0.40***

Table S2 Transcriptional changes in BER, NER and *BDNF* genes during human aging in four different brain Fold change in gene expression in aged individuals (age 69-99 years) compared to young individuals (age 20-59 years) in four different brain regions. EC: Entorhinal cortex; HC: hippocampus; PCG: postcentral gyrus; SFG: superior frontal gyrus. *: p≤0.05; **: p≤0.01; *** p≤0.001; ****: p≤0.0001. Name of probesets in the microarray analysis are displayed. For genes with more than one probeset an average expression value was calculated.

Gene	CRE site	Sequence (5’-3’)
Ape1	Native	CCACTCACCGGTGCCCGGGGCCG**TGACG**TAAGTGCGCCGCGGGTTCGCCAG
	Scrambled	CCACTCACCGGTGCCCGGGGCCG<u>GAGCT</u>TAAGTGCGCCGCGGGTTCGCCAG
Fen1	Native	ACATCATCCCAGAACTAGATGCA**CGTCA**GATGGTGGTCACTAGGAGGCGTG
	Scrambled	ACATCATCCCAGAACTAGATGCA<u>ACCGT</u>GATGGTGGTCACTAGGAGGCGTG
Lig3	Native	TCAGCCTGGGGGCACACCCTCCG**TGACG**TGGCGCCCCCACGGGAGCCCACG
	Scrambled	TCAGCCTGGGGGCACACCCTCCG<u>TAGCG</u>TGGCGCCCCCACGGGAGCCCACG
Neil1	Native	GGCAATATTCCGGCCTGACTGTG**TGACG**TGAGACACCATCTTTAATTAATT
	Scrambled	GGCAATATTCCGGCCTGACTGTG<u>GAGCT</u>TGAGACACCATCTTTAATTAATT
	Methylated	GGCAATATTCCGGCCTGACTGTG**TGA(5-mC)G**TGAGACACCATCTTTAATTAATT
Neil2	Native	TTCCCGGGACCCACCTGGGGCTG**CGTCA**GCCTCCTCCCTGCCCTCTGTAGC
	Scrambled	TTCCCGGGACCCACCTGGGGCTG<u>TCACG</u>GCCTCCTCCCTGCCCTCTGTAGC
Nthl1	Native	AGCAAAGGCAGGAGCTCAAACGG**TGACG**GTCTGCCTTCATCCCACAGGCTC
	Scrambled	AGCAAAGGCAGGAGCTCAAACGG<u>GAGCT</u>GTCTGCCTTCATCCCACAGGCTC
Ogg1	Native	TACAAAGTGTACATGGTGTGGGG**TGACG**ACACACTTAGAATAGCTTTAAGA
	Scrambled	TACAAAGTGTACATGGTGTGGGG<u>GAGCT</u>ACACACTTAGAATAGCTTTAAGA
	Methylated	TACAAAGTGTACATGGTGTGGGG**TGA(5-mC)CG**ACACACTTAGAATAGCTTTAAGA
Polb	Native	AGCCTGGCGCG**TGACGTCA**CCGCGCTGCGC
	Scrambled	AGCCTGGCGCG<u>TAGCCGTA</u>CCGCGCTGCGC
Tdg	Native	AAACTTAGAGCCCACAAAAGC**TGACGTCA**GCCGGGCGCTCCCCATCCTCG
	Scrambled	AAACTTAGAGCCCACAAAAGCC<u>CGGTACTA</u>GCCGGGCGCTCCCCATCCTCG
Ung	Native	AGCTGCACAACTGAATGTATGTC**TGACG**TGAGTGTGGGAGGTAAAAAGTAC
	Scrambled	AGCTGCACAACTGAATGTATGTC<u>TGCGA</u>TGAGTGTGGGAGGTAAAAAGTAC
Xrcc1	Native	GCCCAACCGAGCATGCGCAGTGT**TGACG**TGTGCGCCGGCGCGCCGCGGTTT
	Scrambled	GCCCAACCGAGCATGCGCAGTGT<u>GCGTA</u>TGTGCGCCGGCGCGCCGCGGTTT

Table S3 Probes for electromobility shift assays Sequence corresponds to sequence of the selected region of the promoter on the coding strand. Oligonucleotides were annealed with fully complementary oligonucleotides of equal length (not shown). Sequence marked in bold indicates CRE (full/half) site. Underlined sequence indicates the scrambled sequence. For methylated probes the 5-mC was present at the CpG site in the CRE site on both strands.

Activity	Name of oligomer	Sequence 5’-3’
APE1 incision	THF	ATA TAC CGC GG**(THF)** CGG CCG ATC AAG CTT ATT
	Control	ATA TAC CGC GG**C** CGG CCG ATC AAG CTT ATT
	Complementary	TAT ATG GCG CCG GCC GGC TAG TTC GAA TAA
NEIL incision	5-OHU B11	GCT TAG CTT GGA ATC GTA TCA TGT A**(5-OHU)**A CTC GTG TGC CGT GTA GAC CGT GCC
	Control B11	GCT TAG CTT GGA ATC GTA TCA TGT A**C**A CTC GTG TGC CGT GTA GAC CGT GCC
	B11 complementary	GGC ACG GTC TAC ACG GCA CAA ACA GCC CAC GGA TAC GAT TCC AAG CTA AGC
OGG1 incision	8oxoG	ATA TAC CGC G**(8oxoG)**C CGG CCG ATC AAG CTT ATT
	Control	ATA TAC CGC G**C**C CGG CCG ATC AAG CTT ATT
	Complementary	AAT AAG CTT GAT CGG CCG GCC GCG GTA TAT
Incorporation activity	U-HP:G	ATA TAC CAC GTC GG**U** GAT CCA GTC CTG *CTT TTG* CAG GAC TGG ATC GCC GAC GTG GTA TAT
	HP-control (HP-C)	ATA TAC CAC GTC GG**C** GAT CCA GTC CTG *CTT TTG* CAG GAC TGG ATC GCC GAC GTG GTA TAT

Table S4 Oligonucleotides for BER activity assays Activity indicates which BER enzyme(s) that exhibits activity against the damaged oligonucleotide. B11: 11 nt non-complementary sequence is underlined and gives rise to an 11 nt single-stranded bubble structure after annealing. THF: tetrahydrofuran derivate (AP site analogue with a higher stability than naturally occurring AP site); 5-OHU: 5-hydroxyuracil; 8oxoG: 8-oxoguanine. Italics: hairpin loop.

Primer	Sequence 5’-3’	Target region	PCR cycling conditions
NeuroD LA Fwd<br>NeuroD LA Rev	CTCGCAGGTGCAATATGAATC<br>GCAACTGCATGGGAGTTTTCT	7.2 kb at NeuroD gene	94°C 30s, 25 x (94°C 15s, 55°C 30s, 65°C 10m), 65°C 10m
NeuroD SA Fwd<br>NeuroD SA Rev	CTGCAAAGGTTTGTCCCAGC<br>CTGGTGCAGTCAGTTAGGGG	282 bp at NeuroD gene	95°C 30s, 25 x (95°C 15s, 56°C 30s, 68°C 30s), 72°C 5m
Mito LA Fwd<br>Mito LA Rev	GCCAGCCTGACCCATAGCCATATTAT<br>GAGAGATTTTATGGGTGTATTGCGG	10 kb of mtDNA	94°C 30s, 19 x (94°C 15s, 63°C 30s, 65°C 10m), 65°C 10m
Mito SA Fwd<br>Mito SA Rev	CCCAGCTACTACCATCATTCAAGT<br>GATGGTTTGGGAGATTGGTTGATG	117 bp of mtDNA	95°C 30s, 20 x (95°C 15s, 60°C 30s, 68°C 30s), 72°C 5m
Nuclear SA Fwd<br>Nuclear SA Rev	TGTTGGCTCTGTCTGTCGTC<br>GGAGCAGAACCAACCCAAGT	110 bp of POLB gene	95°C 30s, 30 x (95°C 15s, 60°C 30s, 68°C 30s), 72°C 5m

Table S5 Primer and PCR conditions for long-range PCR SA: small amplicon; LA: long amplicon.

Brain region/ Gene	EC	HC	PCG	SFG
**BER genes**				
APE1	-0.30	0.14	-0.29	-0.39*
ERCC6	-0.12	-0.36	-0.27	-0.57***
ERCC8	-0.18	-0.30	-0.16	-0.25
FEN1	-0.31	-0.43*	-0.42*	-0.52***
LIG1	0.09	0.24	-0.09	-0.16
LIG3	-0.20	-0.22	-0.44*	-0.38*
NEIL1	0.08	0.02	-0.11	0.18
NEIL2	-0.16	0.13	-0.38*	-0.44**
NTHL1	-0.31	-0.28	-0.56**	-0.48**
OGG1	-0.19	0.08	-0.03	0.13
PARP1	-0.09	0.08	-0.21	0.04
PNKP	-0.23	-0.17	-0.40*	-0.18
POLB	-0.37	-0.11	-0.41*	-0.53***
SMUG1	-0.49*	-0.56***	-0.45*	-0.57***
TDG	0.01	-0.30	-0.36*	-0.39*
UNG	0.34	0.66****	0.40*	0.55***
XRCC1	0.03	-0.61***	-0.14	0.03
**NER genes**				
ERCC1	-0.11	-0.11	-0.25	-0.01
ERCC2	-0.38	-0.25	-0.53**	-0.31
ERCC3	-0.19	-0.39	-0.27	-0.26
ERCC4	-0.15	-0.01	0.19	-0.12
ERCC5	0.14	-0.32	0.30	0.37*
ERCC6	-0.12	-0.36	-0.27	-0.57***
ERCC8	-0.18	-0.30	-0.16	-0.25
LIG1	0.09	0.24	-0.09	-0.16
LIG3	-0.20	-0.22	-0.44*	-0.38*
XPA	-0.15	-0.19	-0.20	0.04
XRCC1	0.03	-0.61***	-0.14	0.03

Table S6 Correlation between DNA repair gene expression and age EC: entorhinal cortex (N=39); HC: hippocampus (N=40); PCG: postcentral gyrus (N=43); SFG: superior frontal gyrus (N=48). Individuals aged 20-99 years. Normal distribution was tested by Shapiro-Wilk test. Correlation was tested by Spearman's rank correlation coefficient between BER and NER genes and age as a continuous variable. Benjamini-Hochberg correction for multiple testing. *: p≤0.05; **: p≤0.01; *** p≤0.001; ****: p≤0.0001.

Brain region/ Gene	EC	HC	PCG	SFG
**BER genes**				
APE1	0.44**	0.55**	0.32	0.60***
ERCC6	0.16	-0.04	0.30	0.31
ERCC8	0.34	-0.20	0.02	0.39*
FEN1	0.72****	0.02	0.36	0.66****
LIG1	0.04	-0.14	0.05	0.06
LIG3	0.53**	0.16	0.36	0.46**
NEIL1	0.12	0.07	0.04	-0.16
NEIL2	0.61***	0.41*	0.27	0.62****
NTHL1	0.44**	-0.21	0.29	0.66****
OGG1	-0.37*	0.22	-0.14	-0.26*
PARP1	-0.33	-0.15	0.04	0.15
PNKP	0.50**	0.36	0.47*	0.49**
POLB	0.64***	0.43*	0.48	0.63****
SMUG1	0.60****	-0.04	0.31	0.64****
TDG	0.33	0.06	-0.09	0.03
UNG	-0.59**	0.08	-0.48*	-0.47**
XRCC1	0.27	-0.40*	0.33	0.05
**NER genes**				
ERCC1	-0.09	-0.08	0.34	0.33
ERCC2	0.53**	0.50*	0.44	0.56**
ERCC3	0.31	0.13	0.14	0.23
ERCC4	-0.22	-0.23	0.23	-0.05
ERCC5	-0.32	-0.70****	-0.55**	-0.61****
ERCC6	0.16	-0.04	0.30	0.31
ERCC8	0.34	-0.20	0.02	0.39*
LIG1	0.04	-0.14	0.05	0.06
LIG3	0.53*	0.16	0.36	0.46*
XPA	0.30	-0.57**	-0.10	0.22
XRCC1	0.27	-0.40*	0.33	0.05

Table S7 Correlation between expression of BDNF and DNA repair genes in human brains EC: entorhinal cortex (N=38); HC: hippocampus (N=41); PCG: postcentral gyrus (N=42); SFG: superior frontal gyrus (N=47). Individuals aged 20-99 years. Normal distribution was tested by Shapiro-Wilk test. Partial Spearman's rank correlation co-efficient adjusting for age was computed. Benjamini-Hochberg correction for multiple testing. *: p≤0.05; **: p≤0.01; *** p≤0.001; ****: p≤0.0001.

Gene	Accession	Chrom	Strand	TSS Pos	CRE prediction	CRE Flag	Full Site	Half Site	Conserved CRE
**Human genome**									
APEX1	NM_001641<br>NM_080648<br>NM_080649	Chr14	+	18913417	CRE_NoTATA	H ht		ht_-4613; ht_694; H_-546; ht_-12; ht_478	H_-546_458
APTX	NM_175069<br>NM_175073	Chr9	-	32991626<br>32991606	CRE_NoTATA	f h	f_-7; f_-38<br>f_-27; f_-58	h_166; h_76<br>h_146; h_56	
FEN1	NM_004111	Chr11	+	61335509	CRE_TATA	H ht h		h_-1725; H_586; h_208; ht_241	H_-586_1110
LIG1	NM_000243	Chr19	-	53365372	Others	ht		ht_-1637<br>h_-2866	
LIG3	NM_002311<br>NM_013975	Chr17	+	33453133	CRE_NoTATA	H ht		ht_-354, H_205	H_205_321
NEIL1	NM_024608	Chr15	+	73355226	CRE_TATA	None		ht_-4842; ht_-3648	
NEIL2	NM_145043	Chr8	+	11664665	Others	ht h		ht_-1905; h_-1140	
NTHL1	NM_002528	Chr16	-	2037784	Others	ht h		h_-2; h_-643; ht_-2387; ht_-3432; h_-4676	
OGG1	NM_002542<br>NM_016819<br>NM_016820<br>NM_016821<br>NM_016826/016827/ 016828/016829	Chr3	+	9765704<br><br><br>9766660<br>9766653	Others	ht		HT_-4596; ht_-274<br><br><br>ht_-1230<br>ht_-1223	
PNKP	NM_007254	Chr19	-	55062630	Others	ht		ht_-376; ht_-544; h_-3085	
POLB	NM_002690	Chr8	+	42213397	CRE_NoTATA	F ht	F_-65	ht_-420	F_-65_880
POLG	NM_002693	Chr15	-	87607794	Others	h		h_-236	
SMUG1	NM_014311	Chr12	-	52869024	Others	ht		ht_71; ht_-643	
TDG	NM_003211	Chr12	+	102862106	CRE_NoTATA	H ht h		ht_-4824; ht_-4757; ht_-533; h_-276; H_-188; h_-65	H_-188_473
UNG	NM_003362<br>NM_080911	Chr12	+	107998726<br>107998134	Others	ht h		h_-4708; ht_-4532; ht_-1562; h_-328<br>ht_-4798; h_-4116; ht_-3940; ht_-970; h_264	
XRCC1	NM_006297	Chr19	-	48771555	Others	ht		ht_-182	
**Mouse genome**									
Apex1	NM_009687	Chr14	+	42310020	CRE_NoTATA	H ht h		H_-340; h_-208, ht_241	H_-340_710
Aptx	NM_025545	Chr4	-	40592403	CRE_TATA	f	f_-294	h_-4367; h_899	
Fen1	NM_007999	Chr19	-	9207451	CRE_TATA	HT ht h		ht_-2049; HT_-999, h_-173	HT_-999_141
Lig1	NM_010715	Chr7	+	9022658	Others	None			

Lig3	NM_010716	Chr11	+	83568269	CRE_NoTATA	H ht h		ht_-1884; h_-1649; h_-1529; h_27; H_188; ht_769	H_188_452
Neil1	NM_028347	Chr9	-	57307543	CRE_TATA	ht		ht_-1366; ht_-1271; ht_826	
Nthl1	NM_008743	Chr17	+	23320048	Others	h		ht_-4695; h_-2886	
Ogg1	NM_010957	Chr6	+	114130250	Others	None		ht_-4312	
Pnkp	NM_021549	Chr7	+	33484641	Others	ht h		ht_-3316; ht_-2219; h_-1455; ht_123	
Polb	NM_011130	Chr8	-	21395118	CRE_NoTATA	F ht	F_-35	ht_-1955; ht_-678	F_-35_572
Polg	NM_017462	Chr7	-	67800291	Others	h		h_-168	
Smug1	NM_027885	Chr15	-	103619363	Others	ht		ht_-4413; ht_-149	
Tdg	NM_172552	Chr10	+	82211462	CRE_NoTATA	FH	FH_-206	ht_336; ht_758	
Ung	NM_011677	Chr5	+	111307061	Others	h		h_-3844; h_-1118; h_309	
Xrcc1	NM_009532	Chr7	+	15958672	Others	ht h		h_-4046; ht_-39; h_188	

Table S8 CRE site prediction in promoters of human and mouse RefSeq BER genes The Salk Institute CREB Target Database was used to predict CRE sites in the promoters of BER genes (-5 kb to +1 kb from TSS). Chrom/Strand/TSS position: Chromosomal position of TSS and the coding strand based on mm3 mouse genome assembly (UCSC). CRE prediction: Genes with predicted functional CRE. Genes are divided into CRE_TATA and CRE_NoTATA based on the presence of TATA boxes. The rest of the genes are labelled "Others". CRE Flag: Marker for the types of CREs on the promoter (-3 kb to +300 bp from TSS). F/f: full site. H/h: half site. Uppercase letter: conserved CRE. FH: full site CRE in species studied but only half site in other species. T/t: presence of TATA box less than 300 bp downstream of CRE site. Full/half site: all occurrences of full site (TGACGTCA) or half site (TGACG/CGTCA) CREs in -5kb to +1 kb region of TSS. Number marks position relative to TSS (hg16/mm3). Conserved CRE: conserved full or half site. Last number indicates the distance to closest downstream TATA box.

Primer	Sequence (5’-3’)	Amplicon length (bp)	CRE site
APE1 fwd	GATAGAATAAAATTGGATAAGGAGT	299	-340 (H)
APE1 rev	ATAATTAATCCTCCTAACACCTC		
POLB fwd	TAAATTTGATTTTTTAGTTTTTTTT	148	-35 (F)
POLB rev	AAACCCACACCTAAATCTTACC		
NEIL1 fwd	AGGATGTAGTAGGGTGTAGTAGGGT	210	-1366 (ht)
NEIL1 rev	ACAAACCCAAATTTTTAATTAATTC		
UNG fwd	GGGATTGTTATATTAGAGGGAAGGA	209	-1188 (h)
UNG rev	CCCGAAATATTCCACTTCCCCA		
OGG1 fwd	TTTGGGGAATAAATGATATAGGAAGT	247	-4312 (ht)
OGG1 rev	TAAAAACCACACCCTTTTCTTAAAA		

Table S9 Primers for bisulfite sequencing to study methylation at selected CRE sites in the promoter region CRE site covered by primers is named according to Table S8.
